# Comparison of high versus low frequency cerebral physiology for cerebrovascular reactivity assessment in traumatic brain injury: a multi-center pilot study

**DOI:** 10.1007/s10877-019-00392-y

**Published:** 2019-10-01

**Authors:** Eric P. Thelin, Rahul Raj, Bo-Michael Bellander, David Nelson, Anna Piippo-Karjalainen, Jari Siironen, Päivi Tanskanen, Gregory Hawryluk, Mohammed Hasen, Bertram Unger, Frederick A. Zeiler

**Affiliations:** 1 Department of Clinical Neuroscience, Karolinska Institutet, Stockholm, Sweden; 2 Theme Neuro, Karolinska University Hospital, Stockholm, Sweden; 3 Department of Neurosurgery, University of Helsinki and Helsinki University Hospital, Helsinki, Finland; 4 Department of Physiology and Pharmacology, Section of Perioperative Medicine and Intensive Care, Karolinska Institutet, Stockholm, Sweden; 5 Division of Anesthesiology, Department of Anesthesiology, Intensive Care and Pain Medicine, University of Helsinki and Helsinki University Hospital, Helsinki, Finland; 6 Section of Neurosurgery, Division of Surgery, Rady Faculty of Health Science, University of Manitoba, Winnipeg, Canada; 7 Department of Neurosurgery, King Fahad University Hospital, Imam Abdulrahman Bin Faisal University, Dammam, Saudi Arabia; 8 Section of Critical Care, Department of Medicine, Rady Faculty of Health Sciences, University of Manitoba, Winnipeg, Canada; 9 Biomedical Engineering, Faculty of Engineering, University of Manitoba, Winnipeg, Canada; 10 Department of Human Anatomy and Cell Science, Rady Faculty of Health Sciences, University of Manitoba, Winnipeg, Canada; 11 Division of Anaesthesia, Department of Medicine, Addenbrooke’s Hospital, University of Cambridge, Cambridge, UK

**Keywords:** Autoregulation, Cerebrovascular reactivity, Low-frequency, TBI

## Abstract

Current accepted cerebrovascular reactivity indices suffer from the need of high frequency data capture and export for post-acquisition processing. The role for minute-by-minute data in cerebrovascular reactivity monitoring remains uncertain. The goal was to explore the statistical time-series relationships between intra-cranial pressure (ICP), mean arterial pressure (MAP) and pressure reactivity index (PRx) using both 10-s and minute data update frequency in TBI. Prospective data from 31 patients from 3 centers with moderate/severe TBI and high-frequency archived physiology were reviewed. Both 10-s by 10-s and minute-by-minute mean values were derived for ICP and MAP for each patient. Similarly, PRx was derived using 30 consecutive 10-s data points, updated every minute. While long-PRx (L-PRx) was derived via similar methodology using minute-by-minute data, with L-PRx derived using various window lengths (5, 10, 20, 30, 40, and 60 min; denoted L-PRx_5, etc.). Time-series autoregressive integrative moving average (ARIMA) and vector autoregressive integrative moving average (VARIMA) models were created to analyze the relationship of these parameters over time. ARIMA modelling, Granger causality testing and VARIMA impulse response function (IRF) plotting demonstrated that similar information is carried in minute mean ICP and MAP data, compared to 10-s mean slow-wave ICP and MAP data. Shorter window L-PRx variants, such as L-PRx_5, appear to have a similar ARIMA structure, have a linear association with PRx and display moderate-to-strong correlations (r ~ 0.700, p < 0.0001 for each patient). Thus, these particular L-PRx variants appear closest in nature to standard PRx. ICP and MAP derived via 10-s or minute based averaging display similar statistical time-series structure and co-variance patterns. PRx and L-PRx based on shorter windows also behave similarly over time. These results imply certain L-PRx variants may carry similar information to PRx in TBI.

## Introduction

Cerebrovascular reactivity monitoring in adult traumatic brain injury (TBI) is becoming increasingly common in the critical care management of moderate and severe TBI [1, 2]. Impaired cerebrovascular reactivity has been linked to worse global outcome in adult TBI at 6 months [3–6], and appears independent of current intensive care unit (ICU) therapies for TBI [7, 8].

Various metrics for continuous monitoring of cerebrovascular reactivity have been described in adult TBI, based on the invasive/non-invasive modalities employed for monitoring cranial physiology. The intra-cranial pressure (ICP) based measures have received the most attention, with the pressure reactivity index (PRx) considered the standard by many [9]. PRx is one of the few indices with some experimental literature to support its ability to detect the lower limit of autoregulation during both arterial hypotension and intra-cranial hypertension [10–12]. Furthermore, impaired vascular reactivity, as measured through PRx, has an extensive body of literature supporting its association with poor global outcome [4, 5], cerebral metabolic dysfunction [13, 14] and impaired cerebral oxygen delivery [9, 15].

However, despite the promising nature of PRx, it has limitations. One important limitation is the data required for derivation. PRx is based on the concept that the correlation between slow-wave vasogenic fluctuations in a measure of cerebral blood volume (such as ICP) and a driver of cerebral blood flow (such as mean arterial pressure (MAP)), provide a surrogate measure of cerebral autoregulation [3]. As such, the calculation of standard PRx requires 10-s by 10-s mean values of ICP and MAP, focusing on the slow-wave frequency range of 0.05–0.005 Hz. [16, 17] Most bedside monitors in the ICU employed globally struggle to provide data export at such a frequency, with most providing minute-by-minute data as their highest export frequency. As such, PRx has classically required third party software, and in many cases data up-sampling, to be calculated and properly assessed.

Given this, there has been an attempt to utilize minute-by-minute ICP and MAP data to derive low resolution, or low frequency, versions of PRx, termed long-PRx (L-PRx), with the aim to increase accessibility to such monitoring [18–20]. Such measures have shown moderate correlation with standard PRx, and an association with global outcome in adult TBI, in small populations, and likely capture the low-frequency end of the vasogenic frequency range.

Despite this, there exists uncertainty as to whether minute-by-minute ICP and MAP data behave similarly to higher frequency 10-s mean data, and whether L-PRx carries similar information to PRx over time. Previous works focused mainly on the correlation between large averaged time periods of data, not focusing on the statistical properties of the parent signals. In the absence of a direct gold standard reproducible measure of autoregulation or cerebral blood flow in vivo, we cannot directly compare measures such as PRx and L-PRx to flow or vascular regulation. Thus, we are left with exploring the relationships of these derived signals, their structure statistically and behavior over time, in order to make more definitive comments as to whether L-PRx metrics provide close surrogate measure of PRx and may be employed clinically. As such, the goal of this study was to evaluate the statistical time-series relationships between ICP, MAP and PRx/L-PRx using both data derived from 10-s and minute-based means of ICP and MAP in adult TBI.

## Methods

### Patients

Patient data was accrued retrospectively from three centers with archived high resolution physiologic data, with two from Nordic countries (Helsinki University Hospital,Helsinki, Finland, and the Karolinska University Hospital, Stockholm, Sweden), and one from Canada (Winnipeg Health Sciences Centre). All patients were studied during January 2015 to February 2019. All patients from Stockholm and Helsinki were also part the multi-center Collaborative European Neuro Trauma Effectiveness Research in TBI (CENTER-TBI) study high-resolution ICU sub-study cohort [21]. These patients were prospectively recruited during the periods of January 2015 to December 2017. Patients from Winnipeg were studied as part of an ongoing prospective high-resolution TBI database, started in early 2018.

All patients suffered predominantly from moderate to severe TBI (moderate = Glasgow Coma Score (GCS) 9–12, and severe = GCS of 8 or less). A minority of patients suffered from mild TBI (GCS 13-15), with subsequent early deterioration leading to ICU admission for care and monitoring. All patients in this cohort had invasive ICP monitoring conducted in accordance with the BTF guidelines [22].

### Ethics

Data from Stockholm and Helsinki were collected as part of the CENTER-TBI study which had individual national or local regulatory approval ((Stockholm 2014-11473-31/4, Helsinki 21.10.2014, Dnro ETMK: 95/1801/2014). The CENTER-TBI study (EC grant 602150) has been conducted in accordance with all relevant laws of the EU if directly applicable or of direct effect and all relevant laws of the country where the Recruiting sites were located, including but not limited to, the relevant privacy and data protection laws and regulations (the “Privacy Law”), the relevant laws and regulations on the use of human materials, and all relevant guidance relating to clinical studies from time to time in force including, but not limited to, the ICH Harmonised Tripartite Guideline for Good Clinical Practice (CPMP/ICH/135/95) (“ICH GCP”) and the World Medical Association Declaration of Helsinki entitled “Ethical Principles for Medical Research Involving Human Subjects”. Informed Consent by the patients and/or the legal representative/next of kin was obtained, accordingly to the local legislations, for all patients recruited in the Core Dataset of CENTER-TBI and documented in the e-CRF.

Data from the Winnipeg cohort was collected in accordance with full local health research ethics board approval from the University of Manitoba (H2017:181, H2017:188, and H2019:157).

### Data collection

All patients had demographics prospectively recorded. Similarly, all patients had high frequency digital signals from ICU monitoring recorded throughout their ICU stay, with the goal of initiating recording within 24 h of ICU admission. All digital ICU signals were further processed (see Signal Acquisition/Signal Processing). For the purpose of this study, the following admission demographic variables were retrospectively collected: age, sex, admission Glasgow Coma Scale (GCS—total and motor) and admission pupillary response (bilaterally reactive, unilateral reactive, bilateral unreactive). CENTER-TBI data was accessed/extracted using Opal database software [23] for the Stockholm and Helsinki data.

### Signal acquisition

Arterial blood pressure (ABP) was obtained through either radial or femoral arterial lines connected to pressure transducers (Baxter Healthcare Corp. CardioVascular Group, Irvine, CA, or similar devices). ICP was acquired via an intra-parenchymal strain gauge probe (Codman ICP MicroSensor; Codman & Shurtleff Inc., Raynham, MA), parenchymal fiber optic pressure sensor (Camino ICP Monitor, Integra Life Sciences, Plainsboro, NJ, United States; https://www.integralife.com/) or external ventricular drain (in 3 patients). All signals were recorded using digital data transfer or digitized via an A/D converter (DT9801; Data Translation, Marlboro, MA), where appropriate, sampled at frequency of 100 Hertz (Hz) or higher, using the ICM + software (Cambridge Enterprise Ltd, Cambridge, UK, http://icmplus.neurosurg.cam.ac.uk) or Moberg CNS Monitor (Moberg Research Inc, Ambler, PA, USA) or a combination of both. Signal artifacts were removed using both manual and automated methods prior to further processing or analysis.

### Signal processing

Post-acquisition processing of the above signals was conducted using ICM + . Only the acute ICU physiology was utilized for this study (i.e. the first 5 days after injury). For ICP and MAP, two sets of data were produced. First, 10-s moving averages (updated every 10 s to avoid data overlap) were calculated for all recorded signals: ICP and ABP (which produced MAP). Second, minute-by-minute averages were also created for both ICP and MAP.

Next, we derived PRx and L-PRx variants for each patient. PRx was derived via the moving correlation coefficient between 30 consecutive 10 s mean windows of the parent signals (ICP and MAP), updated every minute. This methodology for PRx determination has been employed for over 20 years, with this metric having been preliminarily validated as a measure of the lower limit of autoregulation in experimental animal models [3]. Finally, L-PRx was derived using minute-by-minute mean values of ICP and MAP, with various window lengths for calculation, updated every minute. The window lengths used for L-PRx were: 5, 10, 20, 30, 40 and 60 min windows. This created the following respective L-PRx variants: L-PRx_5, L-PRx_10, L-PRx_20, L-PRx_30, L-PRx_40, and L-PRx_60; based on previous literature on low-frequency PRx variants in TBI [18–20].

### Statistics

All statistical analysis were conducted using R (R Core Team (2016). R: A language and environment for statistical computing. R Foundation for Statistical Computing, Vienna, Austria. URL https://www.R-project.org/) and XLSTAT (Addinsoft, New York, NY; https://www.xlstat.com/en/) add-on package to Microsoft Excel (Microsoft Office 15, Version 16.0.7369.1323).

For all testing described within, the alpha was set at 0.05 for significance. Given the bounded nature of PRx and L-PRx variables, all cerebrovascular reactivity indices were transformed using a Fisher natural logarithmic transformation. Thus, for all reference to PRx and L-PRx within the manuscript, we are referring to the Fisher transformed indices. Similarly, ICP and MAP were transformed using a logarithmic transformation, and for all reference to ICP and MAP within the manuscript we are referring to log10 transformed values.

We currently lack a direct, reliable and reproducible continuous measure of cerebral autoregulation and cerebral blood flow in vivo in humans. As such, previous studies have tried to validate PRx against the Lassen curve [24], in particular the lower limit of autoregulation, in experimental animal models [10–12]. To date, PRx has the largest supporting evidence as a continuous metric of cerebrovascular reactivity in adult TBI. To validate L-PRx, in the absence of human or animal studies evaluating it against the autoregulatory curve, we must rely on its relationship and behavior with respect to PRx. Doing so requires evaluating the derived signal structures of PRx and L-PRx variants over time statistically, using various time series methodologies (see sub-sections to follow). In theory, if the structure and behavior of L-PRx is similar to PRx, then one could say that L-PRx may provide similar information to PRx. Studies like this may provide preliminary support for the use of L-PRx clinically in TBI.

### ICP and MAP analysis

ICP and MAP were analyzed in both 10-s by 10-s and minute-by-minute mean value data sheets, per patient. The time series characteristics of ICP and MAP were independently evaluated in each patient, using Box-Jenkin’s autoregressive integrative moving average (ARIMA) models [25–27]. First, both ICP and MAP were evaluated for time stationarity and trend using: autocorrelation function (ACF) plots, partial autocorrelation function (PACF) plots, augmented Dickey-Fuller (ADF) test for root trend, and the Kwiatkowski–Phillips–Schmidt–Shin (KPSS) test for trend. All tests confirmed non-stationary ICP and MAP time-series in each patient. As such, a first order differencing was introduced to each, and the above-mentioned tests repeated to demonstrate stationary behavior.

Next, the optimal ARIMA structure for ICP and MAP were derived in both the 10-s and minute mean data sheets, for each patient. The auto.arima function was initially employed to determine the upper order limit for tested ARIMA models. Based on this, autoregressive order (p) was varied from 1 to 4, and the moving average order (q) was varied from 1 to 4, while the integrative order (d) was held at 1. All subsequent permutations of the ARIMA orders were assessed, with the model Akaike Information Criterion (AIC) and Log-Likelihood (LL) recorded for every model, for both ICP and MAP, in every patient. Using the AIC and LL, the optimal ARIMA structures for ICP and MAP variables were compared in the 10-s and minute mean data sheets, with the lowest AIC and highest LL values indicating superior models. As an example, the general Box-Jenkin’s ARMA model for ICP can be expressed as follows:

$${\text{ICP}}_{\text{t}} = {\text{c + }}\varepsilon_{\text{t}} { + }\sum\limits_{i = 1}^{p} {\varphi_{{{\text{t}} - {\text{i}}}} } {\text{ICP}}_{{{\text{t}} - {\text{i}}}} + \sum\limits_{j = 1}^{q} {\theta_{{{\text{t}} - {\text{j}}}} } \varepsilon_{{{\text{t}} - {\text{j}}}}$$

where: c = constant, t = time “t”, i = integer, j = integer, p = autoregressive order, ICP = intra-cranial pressure, q = moving average order,  θ = autoregressive coefficient at time “t–i”,  ϕ = moving average coefficient at time “t–j”,  = error term.

Subsequently, the influence of ICP and MAP on one another over time was assessed via Granger causality using stationary first order difference ICP and MAP data, with both the impact of ICP on MAP, and the impact of MAP on ICP tested. This was tested in both the 10-s and minute mean data sheets, for every patient. Both F-test statistic value and p-values were recorded.

Finally, in order to further evaluate if 10-s or minute based mean data made a difference in the behavior of ICP and MAP over time, we derived multi-variate vector ARIMA (VARIMA) models. Such models explore the behavior of two time series recorded simultaneously over time, and are derived via extending the standard Box-Jenkin’s models to multi-variate systems [25, 27, 28]. Further description on this technique can be found in the references. The vector autoregressive moving average model (VARMA) of first order difference ICP and MAP can be represented by the following formula:

$${\text{Y}}_{\text{t}} = {\text{ C}} + {\text{E}}_{\text{t}} \sum\limits_{i = 1}^{p} {A_{{{\text{t}} - {\text{i}}}} } {\text{Y}}_{{{\text{t}} - {\text{i}}}} + \sum\limits_{j = 1}^{q} {{\text{B}}_{{{\text{t}} - {\text{j}}}} } {\text{E}}_{{{\text{t}} - {\text{j}}}}$$

where: C = constant vector, t = time “t”, i = integer, j = integer, p = VARMA autoregressive order, Y = ICP and MAP vector, q = VARMA moving average order, A = autoregressive coefficient matrix at “t–i”, B = moving average coefficient matrix at time “t–j”, E = error term vector.

We utilized differenced ICP and MAP signals, to eliminate trend and seasonality, and employed basic VARMA models with autoregressive order of 4 and moving average order of 4, based on the findings from individual patient ARIMA models of ICP and MAP, for both the 10-s and minute mean based data, for each patient. The VARMA model autoregressive order of 4 was chosen given the optimal ARIMA models for ICP and MAP in most patients had autoregressive orders of 2, and thus the product of these orders is 4. Taking the product of the ARIMA autoregressive orders for VARMA modelling is one suggested method of ensuring adequate model structure to cover such a multi-variate time series [27]. Similarly, the moving average order for the VARMA model was based on the sum of the optimal moving average orders from the ARIMA modelling [27] of ICP and MAP, with an order of 2 being the most commonly displayed optimal order for ICP and MAP. Given this was a pilot exploratory analysis, this general (4,1,4) VARIMA model structure was employed for each patient. The coefficients derived from these VARMA models were then employed to derive impulse response function (IRF) plots between ICP and MAP. The IRF plots provide a descriptive graphical representation of the impact of ICP on MAP, and MAP on ICP, by using the generated VARIMA model and modelling a one unit orthogonal impulse of one variable on the other, and vice versa [27]. The plots depict how much from baseline one variable fluctuates in response to the orthogonal impulse of the other variable, and how many lags in time it takes to recover back to baseline.

### PRx and L-PRx analysis

Using minute-by-minute updated PRx and L-PRx data, the following analysis was conducted. For each patient, locally weighted scatterplot smoothing (LOESS) function plots were created between PRx and each L-PRx variant, assessing the degree of linearity between each L-PRx metric and the conventional PRx. Next, Pearson correlation coefficients between PRx and L-PRx were determined for each patient, for each variant of L-PRx. Of note, the degree of statistical significance of the linear models and Pearson correlation coefficients are artificially increased secondary to autocorrelation between minute-by-minute updated measures, and violation of the pre-conditions of linearity. As such, both the linear model plots and correlation coefficients reported are for purely descriptive purposes only, and the size of the *p* value or nature of the confidence intervals should be interpreted with caution.

Finally, the optimal ARIMA time-series structure was compared for PRx and all L-PRx variants for each individual patient using the following methodology. First, ACF and PACF plots were produced, and both ADF and KPSS testing were conducted, for all PRx/L-PRx measures, confirming non-stationarity. First order differencing was then undertaken to remove all trend components, confirming stationarity by repeating the above-mentioned plots and testing. Next ARIMA models were built for each PRx and L-PRx variable, keeping the differencing order of 1 (i.e. d = 1), and varying both the autoregressive and moving average orders (i.e. p and q, respectively) from 0 to 4, through all respective permutations. The AIC and LL were then tabulated for each of these models, for every patient. Using the AIC and LL, the optimal ARIMA structures for PRx and L-PRx variables were compared, with the lowest AIC and highest LL values indicating superior models.

### Outcome analysis

We briefly evaluated the association with outcome for PRx and the derived L-PRx metrics. Employing univariate logistic regression, we assessed the association between PRx and L-PRx measures to dichotomized Glasgow Outcome Scale Extended (GOSE) at 6 months post-injury. We evaluated both mortality and favourable/unfavourable outcome (defined as GOSE 5 or above for favourable outcome). Models were assessed by area under the receiver operating curve (AUC), Akaike Information Criterion (AIC) and 95% confidence intervals (95% CI), with alpha set at 0.05 for significance. AUC and 95% CI were determined through a bootstrap resampling methodology, employing 2000 iterations.

## Results

### Patient demographics

**Fig. 1:**
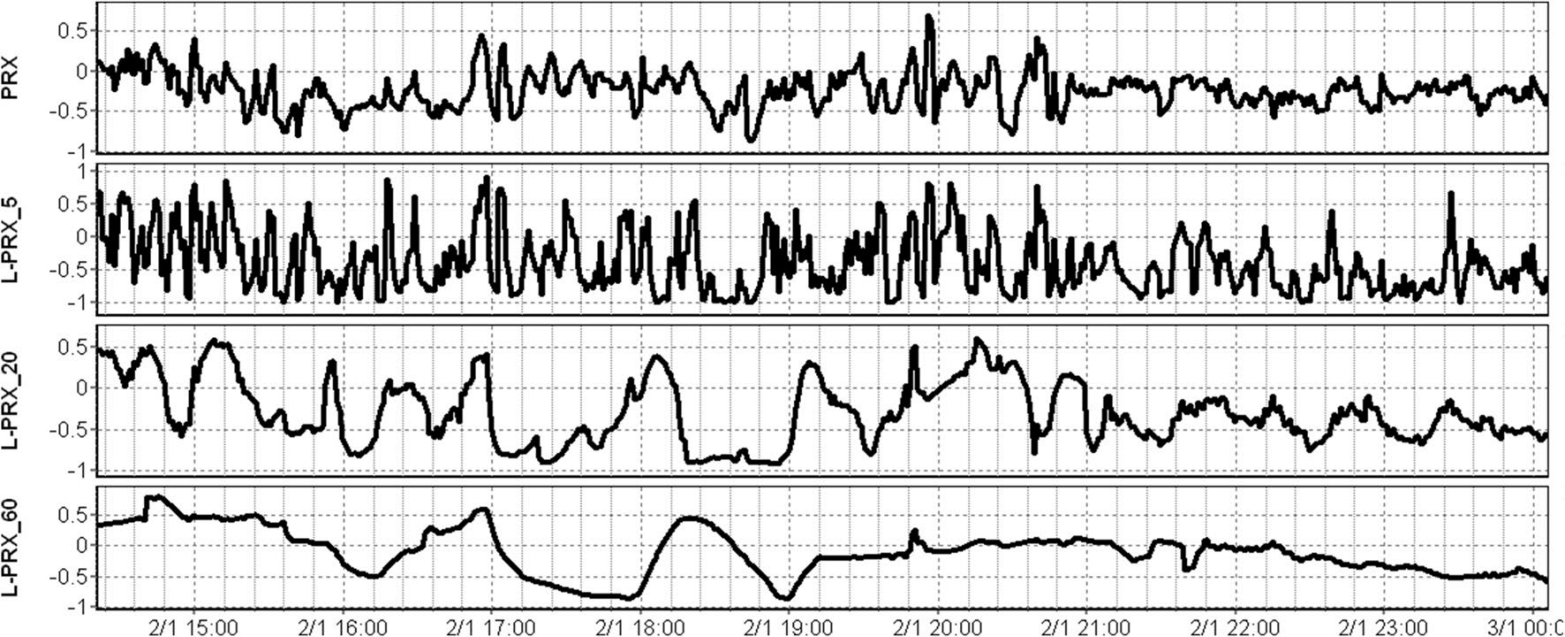
PRx and L-PRx variant behaviour over time—patient example. *ICP* intra-cranial pressure, *MAP* mean arterial pressure, *PRx* pressure reactivity index (correlation between ICP and MAP), *L*-*PRx_5* correlation between minute based ICP and MAP (5-min window length), *L*-*PRx_20* correlation between minute based ICP and MAP (20-min window length), *L*-*PRx_60* correlation between minute based ICP and MAP (60-min window length). Longer window lengths leads to further data smoothing for L-PRx and loss of signal variance in time

A total of 31 patients from the three centers were included in this study. The mean age was 41.7 ± 13.6 years, with 23 (74.2%) male. The median admission total GCS score and motor sub-scores were 6 (IQR: 3 to 8) and 4 (IQR: 1 to 5), respectively. Three patients (9.7%) suffered pre-hospital hypoxic episodes, and three (9.7%) suffered hypotension episodes. Ten patients (32.3%) presented with bilaterally unreactive pupils, and one (3.2%) with unilaterally unreactive pupils. The mean duration of high frequency digital signal recording was 108.3 ± 50.7 h. Table 1 displays the patient admission demographics and injury information. Figure 1 displays a patient example of PRx and some L-PRx variants over time, highlighting the loss of information with larger L-PRx window lengths.

**Table 1:** Admission patient demographics and CT characteristics—median, IQR and raw numbers

		Median (IQR) or raw number
Number of patients		31
Age (years)		43 (30–55)
Sex	Male	23
	Female	8
Duration of high frequency physiologic recording (hours)		120.4 (69.2–138.0)
Admission GCS (total)		6 (3–8)
Admission GCS motor		4 (1–5)
Number with hypoxia episode		3
Number with hypotension episode		3
Admission pupil response	Bilaterally reactive	20
	Unilateral unreactive	1
	Bilaterally unreactive	10
Marshall CT grade	I	0
	II	7
	III	4
	IV	0
	V	5
	VI	18
Number with traumatic SAH		25
Number with epidural hematoma		6
Mean ICP (mmHg)		13.0 (10.8–15.3)
MAP (mmHg)		81.6 (74.7–89.3)
Mean CPP (mmHg)		64.0 (61.6–73.2)
Mean PRx (a.u.)		− 0.052 (− 0.140 to 0.100)
Mean L-PRx_5		− 0.108 (− 0.218 to 0.003)
Mean L-PRx_10		− 0.122 (− 0.221 to 0.028)
Mean L-PRx_20		− 0.098 (− 0.179 to 0.064)
Mean L-PRx_30		− 0.067 (− 0.147 to 0.098)
Mean L-PRx_40		− 0.064 (− 0.120 to 0.100)
Mean L-PRx_60		− 0.021 (− 0.093 to 0.140)
GOSE at 6 months		4 (3–5)
Number dead at 6 months		5
Number unfavrourable outcome at 6 months		16

*a.u.* arbitrary units, *CPP* cerebral perfusion pressure, *CT* computed tomography, *GCS* glasgow coma score, *GOSE* glasgow outcome score, *ICP* intra-cranial pressure, *IQR* inter-quartile range, *IVH* intra-ventricular hemorrhage, *MAP* mean arterial pressure, *mm* millimetres, *mmHg* millimetres of mercury, *PRx* pressure reactivity index (correlation between slow-waves in ICP and MAP), *L*-*PRx* correlation between ICP and MAP using min-by-min average data (the number after L-PRx indicates the calculation window length in minutes), *SAH* subarachnoid hemorrhage

### ICP and MAP analysis—ARIMA structure

We evaluated the ARIMA time-series structure for both ICP and MAP, in every patient, using log transformed and first order differenced data (of note, this transformed and differenced data was utilized for all parts of the ICP and MAP analysis). This was conducted in both 10-s and minute mean data. Table 4 displays a patient example of the AIC and LL for each ARIMA model tested, in both data sheets. In general, the optimal ARIMA structure for ICP and MAP varied between patients, given inter-patient heterogeneities. However, comparing the ARIMA structures of ICP and MAP between the 10-s and minute averaged data, there was no substantial difference in the optimal ARIMA models. This suggests that the time-series behavior is similar for 10-s average vasogenic slow-wave ICP and MAP data, compared to minute-by-minute ICP and MAP data.

### ICP and MAP analysis—granger causality

In order to further assess for differences between 10-s and minute mean data for ICP and MAP (transformed and differenced), we performed Granger causality testing in both sets of data for all individual patients. For most patients, the directional nature of the causal relationship regardless of whether 10-s or minute mean data were assessed, favored MAP impacting ICP. A small minority of patients displayed the alternative causal relationship. Table 2 provides the Granger test results, including F-test and p values, for every patient. Of note, the causal direction of the ICP and MAP relationship was unchanged, regardless of testing on 10-s or minute mean data. This suggests that minute-by-minute mean data does not lose information regarding the ICP and MAP relationship, which is seen in 10-s vasogenic slow-wave data.

**Table 2:** Granger testing for 10-s and minute mean data of ICP and MAP—each patient

Patient	10-s mean data				Minute mean data			
	Granger test: MAP on ICP		Granger test: ICP on MAP		Granger test: MAP on ICP		Granger test: ICP on MAP	
	F-test value	P-value	F-test value	P-value	F-test value	P-value	F-test value	P-value
1	7.325688	P < 0.0001	1.747827	0.13637	7.821262	P < 0.0001	5.854906	0.0001
2	196.5859	P < 0.0001	352.1715	P < 0.0001	9.799218	P < 0.0001	66.01506	P < 0.0001
3	184.9533	P < 0.0001	154.9658	P < 0.0001	7.237617	P < 0.0001	1.699532	0.1480
4	1189.339	P < 0.0001	71.14927	P < 0.0001	100.9624	P < 0.0001	35.47658	P < 0.0001
5	818.9046	P < 0.0001	26.45915	P < 0.0001	34.82038	P < 0.0001	5.414004	0.0002
6	812.6522	P < 0.0001	15.72204	P < 0.0001	4.866256	0.0006	1.214182	0.3024
7	258.3201	P < 0.0001	138.3555	P < 0.0001	4.087783	0.0026	61.22348	P < 0.0001
8	172.6672	P < 0.0001	28.3907	P < 0.0001	37.7531	P < 0.0001	8.736965	P < 0.0001
9	642.1818	P < 0.0001	186.28	P < 0.0001	32.91065	P < 0.0001	6.869269	P < 0.0001
10	384.2702	P < 0.0001	105.4163	P < 0.0001	13.43864	P < 0.0001	26.45632	P < 0.0001
11	236.045	P < 0.0001	115.1936	P < 0.0001	24.51224	P < 0.0001	3.411614	0.0086
12	20.88989	P < 0.0001	23.59018	P < 0.0001	2.348149	0.0526	6.360937	P < 0.0001
13	578.186	P < 0.0001	19.58322	P < 0.0001	7.868772	P < 0.0001	4.15957	0.0023
14	800.0953	P < 0.0001	80.899	P < 0.0001	22.39293	P < 0.0001	4.650688	0.0010
15	249.1448	P < 0.0001	116.2926	P < 0.0001	23.60508	P < 0.0001	8.69085	P < 0.0001
16	531.3783	P < 0.0001	135.6271	P < 0.0001	37.61616	P < 0.0001	3.210749	0.0121
17	968.5263	P < 0.0001	35.87097	P < 0.0001	27.68981	P < 0.0001	3.218454	0.0120
18	114.6648	P < 0.0001	22.43942	P < 0.0001	2.586889	0.0352	1.739461	0.1385
19	3.201506	0.0123	16.05543	P < 0.0001	3.984857	0.0033	1.295173	0.2703
20	91.71564	P < 0.0001	160.2726	P < 0.0001	64.00512	P < 0.0001	75.05347	P < 0.0001
21	148.4782	P < 0.0001	39.81235	P < 0.0001	25.25603	P < 0.0001	17.93121	P < 0.0001
22	85.3137	P < 0.0001	16.24744	P < 0.0001	5.1636	0.0004	17.6889	P < 0.0001
23	1936.034	P < 0.0001	86.56561	P < 0.0001	86.15334	P < 0.0001	9.481133	P < 0.0001
24	596.9424	P < 0.0001	87.43785	P < 0.0001	94.85877	P < 0.0001	3.510931	0.0072
25	96.15071	P < 0.0001	43.77806	P < 0.0001	0.949273	0.4342	4.668226	0.0009
26	862.1427	P < 0.0001	34.35032	P < 0.0001	7.996847	P < 0.0001	54.21212	P < 0.0001
27	126.9935	P < 0.0001	7.208399	P < 0.0001	47.36374	P < 0.0001	17.70014	P < 0.0001
28	247.8369	P < 0.0001	17.96821	P < 0.0001	11.54719	P < 0.0001	1.151579	0.3302
29	226.7049	P < 0.0001	85.50865	P < 0.0001	7.976345	P < 0.0001	10.67541	P < 0.0001
30	1410.132	P < 0.0001	76.97432	P < 0.0001	72.90067	P < 0.0001	8.287524	P < 0.0001
31	1242.374	P < 0.0001	59.30911	P < 0.0001	15.94597	P < 0.0001	2.337457	0.0534

Table displays Granger causality testing for MAP on ICP and ICP on MAP, using 10-s and minute mean data sets. The larger F-test value indicates which direction is favored in the relationship between two variables. In general, the directional nature of causality favors MAP on ICP, using both the 10-s and minute mean data

*ICP* intra-cranial pressure, *MAP* mean arterial pressure

### ICP and MAP analysis—VARMA and impulse response

Finally, to assess the relationship between ICP and MAP further, and whether there was a difference in information carried between 10-s and minute mean data, we employed VARIMA modelling. VARIMA models of autoregressive order 4, integrative order 1, and moving average order 4 were employed for each individual patient, for both the 10-s and minute mean data. IRF plots were produced to provide a descriptive visualization of the relationship between ICP and MAP, in both data sets. These IRF plots allowed us to visually determine the relationship between ICP and MAP, assessing the impact of one unit impulse in MAP on ICP, using transformed data.

**Fig. 2:**
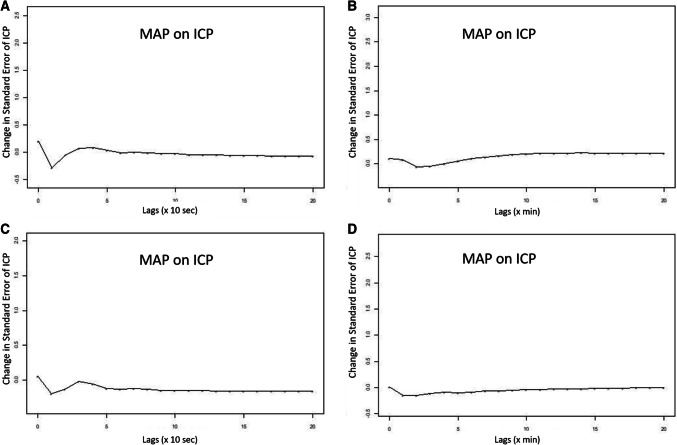
VARIMA impulse response function plots of MAP acting on ICP—two patient examples. *ICP* intra-cranial pressure, *lag* previous measure in time (× 10-s = means 1 lag is 10-s in time; x min = means 1 lag is 1 min in time), *MAP* mean arterial pressure, *VARIMA* vector autoregressive integrative moving average models (for all patients autoregressive order was 4, integrative order 1, and moving average order 4). Panel A and B = Patient 1, and Panel C and D = Patient 2. Plots demonstrate transformed and differenced (i.e. de-trended) minute mean ICP and MAP data (Panel B and D), and 10-s mean data (Panel A and C). Plots display the impact of one standard deviation impulse in MAP on the standard error in ICP from the VARIMA models. Both examples highlight some smoothing in response seen with minute mean data, however the general response in ICP to MAP impulse is similar between minute and 10-s mean data

Overall, using minute mean data led to similar absolute changes in the ICP standard error, with some blunting of the response of ICP to the impulse in MAP, likely related to the data smoothing seen in the minute mean data. This was seen in all patients, and confirms that some information regarding the slow-wave relationship between ICP and MAP is lost in the minute mean data, however there is preservation of the overall general shape of response of ICP to changes in MAP. This, suggests that the minute mean data does indeed contain some information regarding cerebrovascular reactivity. Figure 2 displays the IRF plots for two patient examples, highlighting the similar ICP response to a MAP impulse.

### PRx and L-PRx variants—linear relationships

**Fig. 3:**
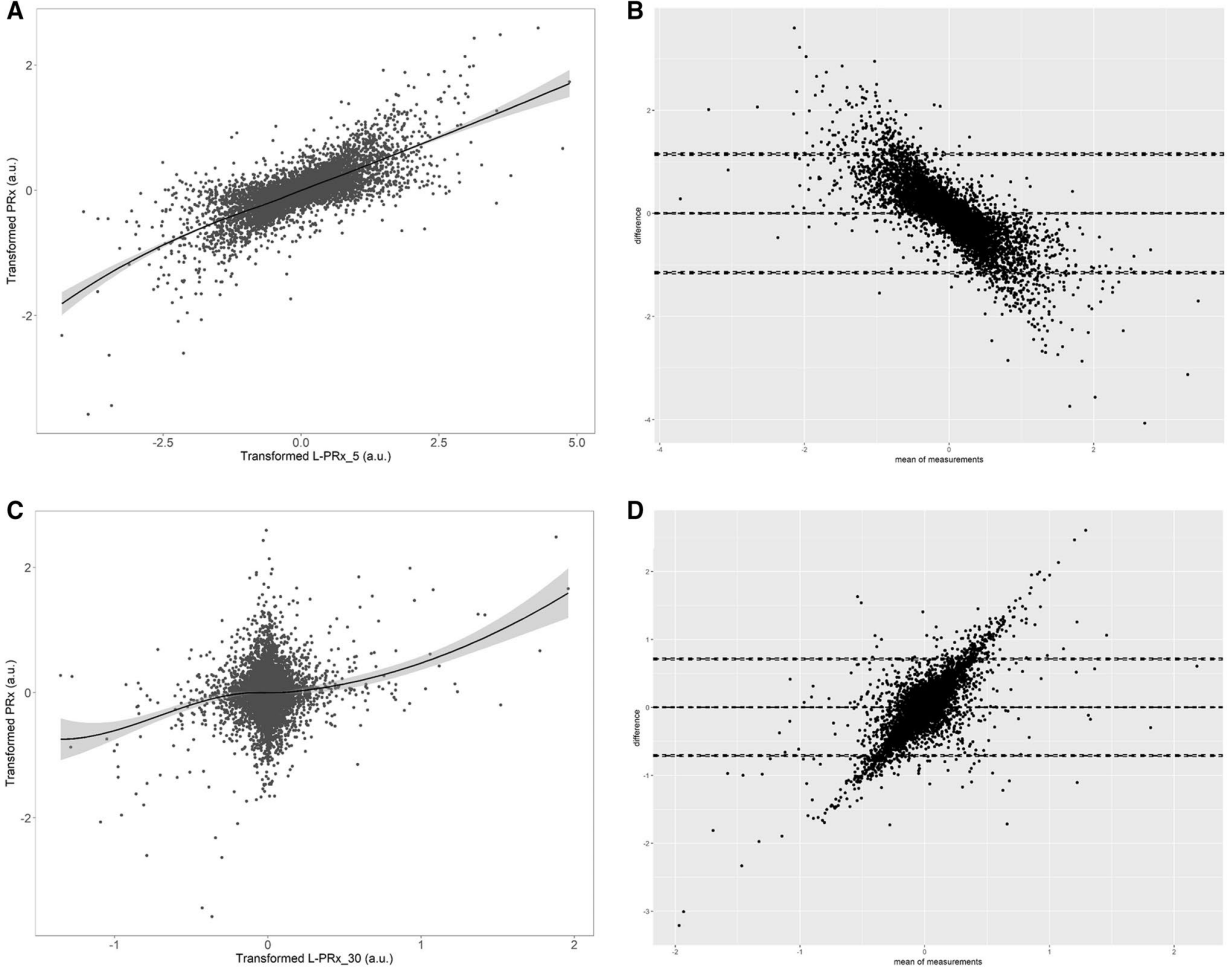
LOESS and Bland–Altman plots for PRx versus L-PRx_5 and PRx versus L-PRx_30—patient example. *a.u.* arbitrary units, *ICP* intra-cranial pressure, *LOESS* locally weighted scatterplot smoothing function, *MAP* mean arterial pressure, *PRx* pressure reactivity index (correlation between ICP and MAP), *L*-*PRx_5* correlation between minute based ICP and MAP (5-min window), *L*-*PRx_30* correlation between minute based ICP and MAP (30-min window)

For each individual patient the linear relationship between PRx and L-PRx variants was assessed using LOESS plots, Pearson correlations and Bland–Altman Analysis. Table 3 provides the Pearson correlation between PRx and the L-PRx variants for every patient. Overall, PRx was most closely associated with L-PRx_5, with the highest Pearson correlation values (~ 0.700, p < 0.0001 for all patients) and visually the strongest linear relationship on LOESS analysis. Similarly, Bland–Altman analysis confirmed in every patient to display a consistent overestimation bias of L-PRx_5. All other L-PRx variants performed poorly in comparison to standard PRx. Figure 3 displays a patient example of LOESS and Bland–Altman plots for PRx versus L-PRx_5, and PRx versus L-PRx_30.

**Table 3:** Pearson correlation testing per patient—PRx versus L-PRx variants

Patient	Pearson correlation coefficients											
	PRx versus L-PRx_5	P	PRx versus L-PRx_10	P	PRx versus L-PRx_20	P	PRx versus L-PRx_30	P	PRx versus L-PRx_40	P	PRx versus L-PRx_60	P
1	0.738	p < 0.0001	0.305	p < 0.0001	0.229	p < 0.0001	0.180	p < 0.0001	0.146	p < 0.0001	0.127	p < 0.0001
2	0.681	p < 0.0001	0.289	p < 0.0001	0.207	p < 0.0001	0.169	p < 0.0001	0.149	p < 0.0001	0.117	p < 0.0001
3	0.667	p < 0.0001	0.280	p < 0.0001	0.200	p < 0.0001	0.178	p < 0.0001	0.131	p < 0.0001	0.114	p < 0.0001
4	0.827	p < 0.0001	0.311	p < 0.0001	0.195	p < 0.0001	0.147	p < 0.0001	0.132	p < 0.0001	0.094	p < 0.0001
5	0.756	p < 0.0001	0.259	p < 0.0001	0.167	p < 0.0001	0.101	p < 0.0001	0.092	p < 0.0001	0.085	p < 0.0001
6	0.660	p < 0.0001	0.290	p < 0.0001	0.217	p < 0.0001	0.163	p < 0.0001	0.131	p < 0.0001	0.117	p < 0.0001
7	0.642	p < 0.0001	0.328	p < 0.0001	0.295	p < 0.0001	0.269	p < 0.0001	0.240	p < 0.0001	0.213	p < 0.0001
8	0.730	p < 0.0001	0.313	p < 0.0001	0.222	p < 0.0001	0.183	p < 0.0001	0.159	p < 0.0001	0.129	p < 0.0001
9	0.661	p < 0.0001	0.302	p < 0.0001	0.209	p < 0.0001	0.155	p < 0.0001	0.129	p < 0.0001	0.086	p < 0.0001
10	0.746	p < 0.0001	0.289	p < 0.0001	0.224	p < 0.0001	0.197	p < 0.0001	0.157	p < 0.0001	0.109	p < 0.0001
11	0.688	p < 0.0001	0.275	p < 0.0001	0.206	p < 0.0001	0.145	p < 0.0001	0.113	p < 0.0001	0.100	p < 0.0001
12	0.737	p < 0.0001	0.276	p < 0.0001	0.262	p < 0.0001	0.225	p < 0.0001	0.155	p < 0.0001	0.123	p < 0.0001
13	0.718	p < 0.0001	0.284	p < 0.0001	0.234	p < 0.0001	0.214	p < 0.0001	0.202	p < 0.0001	0.166	p < 0.0001
14	0.689	p < 0.0001	0.269	p < 0.0001	0.220	p < 0.0001	0.196	p < 0.0001	0.163	p < 0.0001	0.105	p < 0.0001
15	0.703	p < 0.0001	0.269	p < 0.0001	0.174	p < 0.0001	0.157	p < 0.0001	0.123	p < 0.0001	0.087	p < 0.0001
16	0.647	p < 0.0001	0.292	p < 0.0001	0.211	p < 0.0001	0.171	p < 0.0001	0.152	p < 0.0001	0.118	p < 0.0001
17	0.712	p < 0.0001	0.321	p < 0.0001	0.249	p < 0.0001	0.211	p < 0.0001	0.186	p < 0.0001	0.144	p < 0.0001
18	0.682	p < 0.0001	0.280	p < 0.0001	0.164	p < 0.0001	0.111	p < 0.0001	0.070	p < 0.0001	0.066	p < 0.0001
19	0.801	p < 0.0001	0.322	p < 0.0001	0.246	p < 0.0001	0.105	p < 0.0001	0.125	p < 0.0001	0.018	p < 0.0001
20	0.749	p < 0.0001	0.303	p < 0.0001	0.234	p < 0.0001	0.198	p < 0.0001	0.186	p < 0.0001	0.153	p < 0.0001
21	0.720	p < 0.0001	0.254	p < 0.0001	0.149	p < 0.0001	0.121	p < 0.0001	0.095	p < 0.0001	0.054	p < 0.0001
22	0.645	p < 0.0001	0.298	p < 0.0001	0.212	p < 0.0001	0.162	p < 0.0001	0.163	p < 0.0001	0.128	p < 0.0001
23	0.581	p < 0.0001	0.312	p < 0.0001	0.265	p < 0.0001	0.259	p < 0.0001	0.243	p < 0.0001	0.210	p < 0.0001
24	0.793	p < 0.0001	0.274	p < 0.0001	0.1855	p < 0.0001	0.124	p < 0.0001	0.087	p < 0.0001	0.074	p < 0.0001
25	0.702	p < 0.0001	0.311	p < 0.0001	0.223	p < 0.0001	0.184	p < 0.0001	0.145	p < 0.0001	0.105	p < 0.0001
26	0.736	p < 0.0001	0.311	p < 0.0001	0.262	p < 0.0001	0.220	p < 0.0001	0.214	p < 0.0001	0.121	p < 0.0001
27	0.743	p < 0.0001	0.291	p < 0.0001	0.181	p < 0.0001	0.157	p < 0.0001	0.128	p < 0.0001	0.092	p < 0.0001
28	0.713	p < 0.0001	0.291	p < 0.0001	0.209	p < 0.0001	0.154	p < 0.0001	0.129	p < 0.0001	0.099	p < 0.0001
29	0.684	p < 0.0001	0.265	p < 0.0001	0.172	p < 0.0001	0.130	p < 0.0001	0.122	p < 0.0001	0.055	p < 0.0001
30	0.539	p < 0.0001	0.252	p < 0.0001	0.195	p < 0.0001	0.167	p < 0.0001	0.140	p < 0.0001	0.103	p < 0.0001
31	0.441	p < 0.0001	0.202	p < 0.0001	0.150	p < 0.0001	0.135	p < 0.0001	0.126	p < 0.0001	0.089	p < 0.0001

p-values are small given autocorrelation in min-by-min updated data. As such, one can only comment on the magnitude of the Pearson correlation coefficient, not the degree of statistical significance. Also, PRx and L-PRx variants tested were Fisher transformed de-trended (i.e. differenced) variables

*P* p-value, *PRx* pressure reactivity index (correlation between slow waves of ICP and MAP), *L*-*PRx* long PRx (correlation between min-by-min mean data for ICP and MAP; window length for correlation calculation varied, *L*-*PRx_5* 5 min window, etc

### PRx and L-PRx variants—ARIMA structure

Finally, the ARIMA time-series structure for PRx and L-PRx variants were assessed in each patient. Table 5 provides some patient examples of the ARIMA analysis, documenting the ARIMA model, AIC and LL. As with ICP and MAP testing, the optimal ARIMA model for PRx and L-PRx variants, was patient dependent. However, in general, PRx and the small window length versions of L-PRx (i.e. L-PRx_5 and L-PRx_10) had similar ARIMA structures within any individual patient. L-PRx variants with larger window lengths for calculation were increasingly dissimilar to standard PRx based on optimal ARIMA structure, as seen in every patient.

### Outcome association

We performed univariate logistic regression analysis between mean PRx and L-PRx metrics with dichotomized GOSE at 6 months. In keeping with other studies, these PRx/L-PRx metrics displayed stronger association with mortality than favourable/unfavourable outcome [18–20]. The AUC for all PRx and L-PRx measures were not statistically different, with large overlapping 95% CI’s, consistent with such a small cohort. However, despite the limited conclusions that can be made, we can say that the L-PRx metrics display statistically significant associations with mortality at 6 months, as does PRx. All metrics failed to display a significant association with favourble/unfavourable outcome at 6 months. Table 6 provides a tabulated summary of the AUC, 95% CI and AIC values for the univariate logistic regression analysis.

## Discussion

Given the absence of direct, reliable and reproducible metrics of cerebral autoregulation and cerebral blood flow in vivo in adult TBI, to evaluate whether L-PRx is similar to PRx, we must rely on investigating the statistical relationships between high-frequency fluctuations in the derived signals. To do so requires evaluating such behavior over time between both the parent signals used to derive PRx and L-PRx, and also the relationship between PRx and L-PRx metrics themselves. As such, we employed complex time-series analyses via ARIMA, VARMA and Granger causality testing. Through in-depth evaluation of the relationship between ICP and MAP, using 10-s and minute based mean data, and comparing standard PRx to L-PRx variants, some important insights into lower resolution cerebrovascular reactivity metrics have been made. This provides some preliminary evidence to support that certain L-PRx metrics may be considered to be applied clinically in TBI monitoring, after further prospective investigation. Further, these results carry implications to expand the application of cerebrovascular reactivity monitoring, through low-frequency L-PRx metrics, to centers without biomedical signal processing expertise or ability to capture high-frequency full waveform physiology.

First, minute based mean ICP and MAP data displays similar time-series relationships, Granger causality and IRF plots, compared to the conventional 10-s averaged vasogenic slow-wave ICP and MAP data. This is important because despite the smoothing effect seen with minute based ICP and MAP data, and concerns about only capturing the ultra-low end of the vasogenic slow-wave frequency spectrum (i.e. 0.005 Hz or less), minute based data appears to still carry some information regarding the ICP and MAP relationship seen in the higher frequency 10-s data set. This implies that minute based ICP and MAP data may carry information regarding cerebrovascular reactivity, supporting their use in the derivation of L-PRx measures. Though we must acknowledge, the results found in this manuscript are preliminary and require much further validation.

Second, despite both the Granger testing and VARIMA IRF plots indicating that the minute based data likely carries some information regarding cerebrovascular reactivity from the ultra-low end of the vasogenic slow-wave frequency range, there are some subtle findings that also suggest this data carries other potential information. The magnitude of the Granger F-test statistic decreased for the MAP on ICP causal relationship, when evaluating the minute based data, with two patients falling out of significance for this relationship. Thus, despite the causal direction between ICP and MAP remaining in favor of MAP on ICP, when going from 10-s to minute-based data, there is suggestion that this relationship is less strong. This likely stems from the fact that minute-based data captures information on the MAP and ICP relationship outside of the ultra-low end of the vasogenic slow-wave frequency range associated with cerebral autoregulation [16, 17]. Similarly, the IRF plots for MAP on ICP also hint that there is information regarding the relationship of MAP on ICP that extends beyond the vasogenic range. This is highlighted by the ICP response to MAP in the IRF plots taking on the order of minutes to return to baseline in minute based data, where the 10-s data returns to baseline after a minute or two. However, even though these results suggest that minute-based data carries additional low-frequency information regarding the MAP and ICP relationship, beyond the ultra-low end of the vasogenic slow-wave frequency range (i.e. 0.005 Hz), it is unclear at this time what this represents. Much further work is required in this area.

Third, L-PRx variants display heterogeneous relationships with standard PRx. This is in parallel to previous retrospective analysis on low frequency cerebrovascular reactivity metrics derived from minute-by-minute data [19]. Based on Pearson correlation, the correlation between standard PRx and L-PRx_5 was moderate to strong, with coefficients around 0.7–0.8 for most patients in this cohort. The lack of stronger correlation may be related to deriving the Pearson value on 5 paired measures of ICP and MAP for L-PRx_5. However, of importance is the stronger relationship between L-PRx metrics based on short window durations with standard PRx. This was seen in the ARIMA time-series analysis and both LOESS and correlation testing. This implies that if one were to use L-PRx as a measure of cerebrovascular reactivity, that shorter window lengths for calculation should be used, as opposed to the longer windows which appear to have little association with PRx derived from 10-s mean slow-wave data. This should not be a complete surprise, as longer window lengths for L-PRx calculation utilize longer periods of data recording than standard PRx or shorter window L-PRx variants. As such, longer L-PRx metrics are based on increasingly different data, as the window length increases. The reason for including such longer variants was to highlight the substantial difference compared to standard PRx, as these have been evaluated as low-frequency cerebrovascular reactivity metrics in the past [19, 20]. Again, our population was small, and further investigation in larger cohorts is required to validate the findings here.

Fourth, it must be re-emphasized that our results do not absolutely confirm or refute L-PRx metrics as measures of cerebrovascular reactivity. We only demonstrate preliminarily that aspects of L-PRx derivation and specific L-PRx metrics may carry similar information as standard PRx. This is important, since widespread adoption of continuous cerebrovascular reactivity monitoring is hindered by the equipment and expertise required for high-frequency data capture and derivation of standard PRx based on vasogenic slow-wave frequency data. Our results suggest a potential role for L-PRx measures as a surrogate for cerebrovascular reactivity monitoring, in the absence of the ability to derive PRx. Our results require much further validation and exploration in larger adult TBI data sets, as well as in experimental animal models. Given the results of this multi-center pilot analysis, we plan on future large prospective data collection to explore these findings in more detail, integrating patient, injury and treatment characteristics.

Finally, briefly evaluating the association between PRx and L-PRx and 6 month dichotomized GOSE demonstrated corollary findings that L-PRx displays similar outcome association to PRx. This is in keeping with previously published retrospective studies [18–20]. Our results must be interpreted with caution however, given the small cohort size. This is reflected in wide 95% CI’s. As such, at this time we cannot say one metric is superior to the other, or if the lack of association between PRx and L-PRx with favourable/unfavourable outcome is true. This will require much larger prospective studies, which we have planned.

In summary, these results provide some support for low-frequency physiology in the derivation and monitoring of cerebrovascular reactivity in adult TBI. With further prospective evaluation of such metrics, L-PRx provides the ability for a larger number of centers globally to be able to monitor cerebrovascular reactivity in their critically ill cohorts, without the need for specialized biomedical signal processing capabilities, or high-frequency digital data, which is often not available in many centers. The results here imply that low-frequency physiology below the low end of the vasogenic slow wave spectrum (i.e. 0.005 Hz and below), provides at least some information regarding cerebrovascular reactivity. This does not mean it provides all, or even the most optimal, amount of information regarding cerebrovascular reactivity in TBI. The current literature body supports that the optimal slow-wave frequency range for vascular reactivity monitoring existing between 0.05 and 0.005 Hz, as demonstrated in animal models interrogating the lower-limit of autoregulation during arterial hypotension [16, 17]. This frequency range is what PRx, and similar metrics, are designed to capture. As such, when the opportunity to monitor cerebrovascular reactivity based on high-frequency physiologic data exists, this should be the goal as it provides the ability to use a vascular reactivity metric, such as PRx, which is derived in a manner to capture this frequency range. L-PRx should only be considered in the setting where there is a lack of specialized expertise to derive PRx, or there is simply no ability to obtain higher frequency ICP and ABP physiology. The latter is often the case in many centers, given limitations in data export options for commercially available ICU monitors. These are the situations where L-PRx carries a potential to improve access to cerebrovascular reactivity monitoring.

## Limitations

Despite the promising results found, there are some limitations which deserve highlighting. First, this is retrospective analysis of prospectively collected data, in a small data set. As such, our findings can only be considered exploratory and preliminary in nature. The 31 patients were selected based on these being the only TBI patients from the 3 centers with available ICM + data for analysis. As such, the results here may not be generalizable to other TBI populations, necessitating much needed validation. Second, patient, injury and treatment heterogeneity could have influenced the physiologic signal response, and is something that can only be accounted for in larger prospective multi-center data sets. This can be highlighted by the fact that 3 of the 31 patients had EVD’s. Though in this particular cohort there was no difference found in the relationship between PRx and L-PRx, compared to the non-EVD cohort, when looking at the individual patient level, there exists the potential that such differences in monitoring devices, and therapies given, may impact the statistical signal properties. As such, the results here should be interpreted as preliminary, requiring much further validation prior to widespread adoption of L-PRx for clinical monitoring in adult TBI. Finally, the statistical methodology employed was computationally tasking. Larger prospective studies will benefit from more robust central computing services in order to accomplish analysis in a timely fashion.

## Conclusions

ICP and MAP derived via 10-s or minute based averaging display similar time-series statistical structure and co-variance patterns. PRx and L-PRx also behave similarly over time, with those L-PRx indices derived from longer time-windows displaying less association with standard PRx derived from higher frequency data. These results imply certain short window L-PRx variants may carry similar information to PRx in TBI.

## Appendix

See Tables 4, 5 and 6.

**Table 4:** PRx and L-PRx variant ARIMA structure—10 patient examples

ARIMA Model	PRx ARIMA AIC	PRx ARIMA LL	L-PRx_5 ARIMA AIC	L-PRx_5 ARIMA LL	L-PRx_10 ARIMA AIC	L-PRx_10 ARIMA LL	L-PRx_20 ARIMA AIC	L-PRx_20 ARIMA LL	L-PRx_30 ARIMA AIC	L-PRx_30 ARIMA LL	L-PRx_40 ARIMA AIC	L-PRx_40 ARIMA LL	L-PRx_60 ARIMA AIC	L-PRx_60 ARIMA LL
Patient 1														
(0,1,0)	5524.965	− 2760.48	16688.09	− 8342.05	5746.22	− 2871.11	− 2530.24	1267.119	− 6994.37	3499.183	− 10933	5468.507	− 15165.5	7584.757
(0,1,1)	5434.31	− 2714.16	16678.45	− 8336.22	5525.212	− 2759.61	− 3108.66	1557.329	− 7689.33	3847.665	− 11833.5	5919.771	− 16563.8	8284.881
(0,1,2)	5299.259	− 2645.63	16149.73	− 8070.86	5525.939	− 2758.97	− 3173.48	1590.741	− 7783.55	3895.777	− 11962.1	5985.051	− 16712	8359.986
(0,1,3)	4782.03	− 2386.02	15575.44	− 7782.72	5506.07	− 2748.03	− 3172.65	1591.326	− 7793.8	3901.899	− 11972.5	5991.266	− 16721.2	8365.606
(0,1,4)	4509.974	− 2248.99	15322.46	− 7655.23	5455.864	− 2721.93	− 3171.02	1591.51	− 7792.13	3902.067	− 11978.2	5995.106	− 16728.9	8370.466
(1,1,0)	5458.762	− 2726.38	16682.36	− 8338.18	5530.84	− 2762.42	− 3171.83	1588.915	− 7793.72	3899.86	− 11983.6	5994.786	− 16719.5	8362.73
(1,1,1)	5412.346	− 2702.17	15505.94	− 7748.97	5526.46	− 2759.23	− 3170.07	1589.034	− 7795.59	3901.797	− 11984	5995.98	− 16732.9	8370.434
(1,1,2)	4446.258	− 2218.13	15365.91	− 7677.96	5524.692	− 2757.35	− 3172.47	1591.235	− 7794.2	3902.1	− 11982.7	5996.336	− 16734	8372.015
(1,1,3)	4404.948	− 2196.47	15341.47	− 7664.74	4940.662	− 2464.33	− 3170.41	1591.204	− 7792.27	3902.133	− 11984.1	5998.063	− 16741.1	8376.529
(1,1,4)	4406.867	− 2196.43	15320.6	− 7653.3	4930.666	− 2458.33	− 3169.21	1591.607	− 7790.29	3902.146	− 11983.2	5998.622	− 16740.3	8377.161
(2,1,0)	5277.498	− 2634.75	16504.75	− 8248.38	5521.916	− 2756.96	− 3170.11	1589.057	− 7795.79	3901.897	− 11983.9	5995.934	− 16731.4	8369.698
(2,1,1)	4416.414	− 2203.21	15348.21	− 7669.11	4936.18	− 2463.09	− 3169.26	1589.63	− 7794.01	3902.006	− 11981.7	5995.858	− 16731.5	8370.753
(2,1,2)	4417.803	− 2202.9	15347.77	− 7667.88	4918.63	− 2453.32	− 3362	1686.998	− 7792.04	3902.022	− 11980	5995.99	− 16737.8	8374.887
(2,1,3)	4406.922	− 2196.46	15340.33	− 7663.17	4919.785	− 2452.89	− 3169.07	1591.536	− 7790.38	3902.192	− 11982.6	5998.311	− 16739.8	8376.907
(2,1,4)	4408.369	− 2196.18	15319.73	− 7651.86	4921.824	− 2452.91	− 3450.66	1733.33	− 7788.45	3902.226	− 11980.7	5998.36	− 16737.7	8376.847
(3,1,0)	5051.032	− 2520.52	16246.9	− 8118.45	5491.029	− 2740.51	− 3173.54	1591.77	− 7794.15	3902.073	− 11982.2	5996.077	− 16734.8	8372.421
(3,1,1)	4417.471	− 2202.74	15346.22	− 7667.11	4921.187	− 2454.59	− 3449.76	1730.879	− 7792.04	3902.018	− 11979.9	5995.942	− 16740.5	8376.234
(3,1,2)	4419.209	− 2202.6	15350.56	− 7668.28	4933.748	− 2459.87	− 3449.51	1731.753	− 7790.09	3902.043	− 11980.5	5997.238	− 16740.5	8377.269
(3,1,3)	4392.493	− 2188.25	15311.66	− 7647.83	4922.618	− 2453.31	− 3447.19	1731.596	− 7788.39	3902.193	− 11980.9	5998.458	− 16738.6	8377.314
(3,1,4)	4394.471	− 2188.24	15311.25	− 7646.63	4923.049	− 2452.52	− 3448.14	1733.071	− 7786.46	3902.229	− 11978.9	5998.461	− 16736.2	8377.114
(4,1,0)	4947.891	− 2467.95	15982.24	− 7985.12	5461.405	− 2724.7	− 3172.89	1592.443	− 7792.46	3902.23	− 11985.4	5998.691	− 16742.1	8377.07
(4,1,1)	4393.498	− 2189.75	15337.36	− 7661.68	4920.661	− 2453.33	− 3170.52	1592.258	− 7790.48	3902.239	− 11983.8	5998.877	− 16741	8377.475
(4,1,2)	4389.736	− 2186.87	15348.62	− 7666.31	4925.048	− 2454.52	− 3445.79	1730.894	− 7788.47	3902.234	− 11981.5	5998.73	− 16738.8	8377.382
(4,1,3)	4385.492	− 2183.75	15332.41	− 7657.2	4922.839	− 2452.42	− 3445.85	1731.924	− 7964.59	3991.297	− 11981.8	5999.899	− 16737	8377.481
(4,1,4)	4396.491	− 2188.25	15304.73	− 7642.37	4924.171	− 2452.09	− 3444.4	1732.202	− 7960.28	3990.141	− 11979.9	5999.948	− 16734.6	8377.324

ARIMA Model	PRx ARIMA AIC	PRx ARIMA LL	L-PRx_5 ARIMA AIC	L-PRx_5 ARIMA LL	L-PRx_10 ARIMA AIC	L-PRx_10 ARIMA LL	L-PRx_20 ARIMA AIC	L-PRx_20 ARIMA LL	L-PRx_30 ARIMA AIC	L-PRx_30 ARIMA LL	L-PRx_40 ARIMA AIC	L-PRx_40 ARIMA LL	L-PRx_60 ARIMA AIC	L-PRx_60 ARIMA LL
Patient 2														
(0,1,0)	− 749.589	376.7946	24375.14	− 12185.6	3915.933	− 1955.97	− 11869.3	5936.635	− 21450.3	10727.15	− 27503.1	13753.53	− 35386.8	17695.4
(0,1,1)	− 831.138	418.5691	24290.63	− 12142.3	3678.954	− 1836.48	− 12838.6	6422.306	− 22982.9	11494.45	− 29404.7	14705.37	− 37664.3	18835.16
(0,1,2)	− 964.295	486.1474	23687.43	− 11839.7	3679.087	− 1835.54	− 13003.6	6505.793	− 23351	11679.49	− 29823.8	14915.89	− 38284.7	19146.33
(0,1,3)	− 1782.83	896.4129	22662.9	− 11326.5	3654.294	− 1822.15	− 13036.5	6523.247	− 23425	11717.49	− 29958.6	14984.28	− 38430	19220
(0,1,4)	− 2361.56	1186.779	22173	− 11080.5	3565.045	− 1776.52	− 13035	6523.483	− 23452.2	11732.1	− 29995.2	15003.61	− 38473.6	19242.81
(1,1,0)	− 815.216	410.6082	24312.64	− 12153.3	3680.952	− 1837.48	− 13004.9	6505.451	− 23394.3	11700.13	− 29949.6	14977.8	− 38446.5	19226.23
(1,1,1)	− 851.016	429.508	22358.55	− 11175.3	3679.887	− 1835.94	− 13027.6	6517.822	− 23461.5	11734.76	− 30024.9	15016.47	− 38536.3	19272.13
(1,1,2)	− 2463.6	1236.799	22241.61	− 11115.8	3677.744	− 1833.87	− 13030.8	6520.413	− 23460.5	11735.27	− 30023.7	15016.87	− 38534.7	19272.36
(1,1,3)	− 2565.27	1288.636	22185.86	− 11086.9	2779.692	− 1383.85	− 13034.8	6523.411	− 23458.6	11735.3	− 30021.9	15016.94	− 38540.9	19276.46
(1,1,4)	− 2595.42	1304.71	22126.47	− 11056.2	2753.391	− 1369.7	− 13032.7	6523.357	− 23457.9	11735.94	− 30020	15016.98	− 38538.9	19276.47
(2,1,0)	− 969.362	488.681	24150.18	− 12071.1	3677.484	− 1834.74	− 13030.4	6519.217	− 23462.3	11735.16	− 30019.3	15013.63	− 38533	19270.52
(2,1,1)	− 2557.14	1283.568	22216.56	− 11103.3	2790.627	− 1390.31	− 13028.8	6519.403	− 23460.6	11735.3	− 30023.7	15016.83	− 38535.1	19272.55
(2,1,2)	− 2583.07	1297.537	22174.9	− 11081.4	2727.727	− 1357.86	− 13450.1	6731.057	− 23458.9	11735.43	− 30021.8	15016.9	− 38532.4	19272.19
(2,1,3)	− 2592.15	1303.077	22154.1	− 11070	2729.366	− 1357.68	− 13448.8	6731.416	− 23456.9	11735.46	− 30019.8	15016.91	− 38538.2	19276.1
(2,1,4)	− 2601.11	1308.554	22123.1	− 11053.6	2730.454	− 1357.23	− 13446.8	6731.406	− 23455.7	11735.87	− 30017.9	15016.94	− 38537.7	19276.83
(3,1,0)	− 1284.57	647.286	23876.33	− 11933.2	3636.45	− 1813.23	− 13029.3	6519.649	− 23460.6	11735.29	− 30023.7	15016.84	− 38532.6	19271.3
(3,1,1)	− 2589.58	1300.79	22161.75	− 11074.9	2740.434	− 1364.22	− 13026.6	6519.317	− 23458.5	11735.23	− 30021	15016.52	− 38529.6	19270.79
(3,1,2)	− 2588.31	1301.153	22161.7	− 11073.8	2729.397	− 1357.7	− 13029.5	6521.746	− 23457	11735.48	− 30019.7	15016.87	− 38534.8	19274.39
(3,1,3)	− 2583.51	1299.753	22129.78	− 11056.9	2731.636	− 1357.82	− 13448.4	6732.179	− 23454.7	11735.37	− 30017.8	15016.91	− 38541.4	19278.71
(3,1,4)	− 2623.45	1320.724	22116.93	− 11049.5	2733.364	− 1357.68	− 13446.7	6732.337	− 23453.6	11735.82	− 30015.9	15016.93	− 38536.5	19277.23
(4,1,0)	− 1584.97	798.4874	23417.45	− 11702.7	3579.155	− 1783.58	− 13040.9	6526.461	− 23458.9	11735.44	− 30021.8	15016.92	− 38537.9	19274.94
(4,1,1)	− 2589.17	1301.585	22158.26	− 11072.1	2731.162	− 1358.58	− 13448.1	6731.062	− 23457.1	11735.54	− 30019.8	15016.9	− 38539.2	19276.58
(4,1,2)	− 2586.03	1301.016	22150.68	− 11067.3	2730.674	− 1357.34	− 13439.7	6727.874	− 23455.1	11735.57	− 30017.7	15016.83	− 38541.7	19278.87
(4,1,3)	− 2584.61	1301.306	22118.66	− 11050.3	2733.389	− 1357.69	− 13446.6	6732.279	− 23453.6	11735.78	− 30015.8	15016.92	− 38535.9	19276.96
(4,1,4)	− 2608.55	1314.277	22118.81	− 11049.4	2734.729	− 1357.36	− 13443	6731.509	− 23451.7	11735.83	− 30013.8	15016.92	− 38534.8	19277.42

ARIMA Model	PRx ARIMA AIC	PRx ARIMA LL	L-PRx_5 ARIMA AIC	L-PRx_5 ARIMA LL	L-PRx_10 ARIMA AIC	L-PRx_10 ARIMA LL	L-PRx_20 ARIMA AIC	L-PRx_20 ARIMA LL	L-PRx_30 ARIMA AIC	L-PRx_30 ARIMA LL	L-PRx_40 ARIMA AIC	L-PRx_40 ARIMA LL	L-PRx_60 ARIMA AIC	L-PRx_60 ARIMA LL
Patient 3														
(0,1,0)	− 195.15	99.57496	1849.093	− 922.546	407.6061	− 201.803	− 696.589	350.2947	− 1315.62	659.8114	− 1706.26	855.1309	− 2329.14	1166.57
(0,1,1)	− 221.271	113.6354	1845.423	− 919.712	367.3712	− 180.686	− 850.359	428.1797	− 1486.81	746.4065	− 1934.1	970.0511	− 2571.78	1288.891
(0,1,2)	− 220.57	114.2852	1809.81	− 900.905	366.7284	− 179.364	− 869.928	438.9639	− 1523.79	765.8938	− 2024.04	1016.021	− 2660.84	1334.422
(0,1,3)	− 275.435	142.7174	1745.305	− 867.652	368.5118	− 179.256	− 874.432	442.2158	− 1532.55	771.2747	− 2050.29	1030.147	− 2695.18	1352.592
(0,1,4)	− 310.053	161.0266	1698.62	− 843.31	362.4625	− 175.231	− 872.493	442.2464	− 1534.11	773.0574	− 2052.57	1032.283	− 2706.76	1359.378
(1,1,0)	− 216.809	111.4047	1846.807	− 920.403	365.648	− 179.824	− 876.655	441.3274	− 1539.41	772.7031	− 2051.35	1028.674	− 2701.98	1353.991
(1,1,1)	− 219.553	113.7767	1708.012	− 850.006	367.527	− 179.764	− 874.687	441.3436	− 1540.95	774.4743	− 2060.19	1034.093	− 2713.78	1360.89
(1,1,2)	− 324.502	167.2512	1702.025	− 846.013	368.6806	− 179.34	− 872.938	441.4692	− 1539.25	774.6249	− 2061.04	1035.518	− 2713.15	1361.573
(1,1,3)	− 339.21	175.6049	1699.684	− 843.842	298.7192	− 143.36	− 872.471	442.2354	− 1537.52	774.7625	− 2059.42	1035.708	− 2711.32	1361.658
(1,1,4)	− 337.215	175.6075	1696.6	− 841.3	294.0291	− 140.015	− 870.434	442.2169	− 1535.53	774.7658	− 2057.76	1035.878	− 2711.57	1362.785
(2,1,0)	− 230.422	119.211	1838.067	− 915.033	367.452	− 179.726	− 874.69	441.3451	− 1540.68	774.3394	− 2061.91	1034.957	− 2714.97	1361.486
(2,1,1)	− 335.836	172.9181	1700.49	− 845.245	295.6492	− 142.825	− 872.667	441.3337	− 1539.39	774.6962	− 2060.9	1035.451	− 2713.13	1361.563
(2,1,2)	− 333.94	172.9698	1700.198	− 844.099	292.3148	− 140.157	− 910.036	461.0178	− 1537.02	774.5092	− 2059.22	1035.61	− 2711.16	1361.578
(2,1,3)	− 337.214	175.6071	1700.175	− 843.087	294.2399	− 140.12	− 871.375	442.6873	− 1535.54	774.7684	− 2057.07	1035.536	− 2709.15	1361.575
(2,1,4)	− 335.229	175.6147	1698.343	− 841.172	295.6453	− 139.823	− 869.91	442.9551	− 1533.55	774.7767	− 2055.85	1035.923	− 2707.65	1361.826
(3,1,0)	− 266.616	138.3082	1820.191	− 905.096	364.8176	− 177.409	− 873.128	441.5641	− 1539.01	774.5042	− 2061.1	1035.55	− 2713.14	1361.57
(3,1,1)	− 334.008	173.004	1699.027	− 843.514	292.4748	− 140.237	− 909.856	460.9281	− 1537.4	774.6976	− 2059.1	1035.55	− 2711.08	1361.541
(3,1,2)	− 335.307	174.6534	1701.01	− 843.505	294.2614	− 140.131	− 906.32	460.1602	− 1535.51	774.7552	− 2057.75	1035.876	− 2710.21	1362.106
(3,1,3)	− 335.07	175.5348	1698.294	− 841.147	294.3849	− 139.192	− 907.647	461.8233	− 1533.56	774.7808	− 2057.73	1036.867	− 2714.16	1365.079
(3,1,4)	− 333.599	175.7993	1700.139	− 841.069	295.9189	− 138.959	− 905.81	461.9052	− 1531.56	774.7797	− 2055.88	1036.941	− 2705.19	1361.593
(4,1,0)	− 274.565	143.2827	1783.542	− 885.771	357.2933	− 172.647	− 873.835	442.9177	− 1537.43	774.7163	− 2059.1	1035.55	− 2711.15	1361.575
(4,1,1)	− 339.222	176.6108	1700.964	− 843.482	294.1451	− 140.073	− 908.491	461.2455	− 1535.33	774.6665	− 2057.18	1035.588	− 2709.17	1361.585
(4,1,2)	− 337.94	176.97	1701.334	− 842.667	293.9223	− 138.961	− 906.807	461.4036	− 1533.57	774.7874	− 2057.84	1036.922	− 2717.89	1366.947
(4,1,3)	− 333.32	175.6598	1698.809	− 840.405	295.9095	− 138.955	− 906.009	462.0043	− 1531.55	774.7763	− 2055.87	1036.936	− 2715.83	1366.913
(4,1,4)	− 335.135	177.5676	1700.791	− 840.396	297.8667	− 138.933	− 902.85	461.4248	− 1531.84	775.9216	− 2053.77	1036.887	− 2712.21	1366.104

ARIMA Model	PRx ARIMA AIC	PRx ARIMA LL	L- PRx_5 ARIMA AIC	L- PRx_5 ARIMA LL	L-PRx_10 ARIMA AIC	L-PRx_10 ARIMA LL	L-PRx_20 ARIMA AIC	L-PRx_20 ARIMA LL	L-PRx_30 ARIMA AIC	L-PRx_30 ARIMA LL	L-PRx_40 ARIMA AIC	L-PRx_40 ARIMA LL	L-PRx_60 ARIMA AIC	L-PRx_60 ARIMA LL
Patient 4														
(0,1,0)	5493.731	− 2744.87	14996.14	− 7496.07	3739.245	− 1867.62	− 5422.34	2713.168	− 10538.2	5271.124	− 14506.2	7255.119	− 20027.2	10015.58
(0,1,1)	5273.064	− 2633.53	14998.11	− 7496.05	3287.878	− 1640.94	− 6361.54	3183.769	− 11905.4	5955.676	− 16360.6	8183.276	− 22269.8	11137.91
(0,1,2)	5184.694	− 2588.35	14654.69	− 7323.34	3279.012	− 1635.51	− 6488.11	3248.054	− 12067	6037.513	− 16640	8324.024	− 22660.9	11334.43
(0,1,3)	4682.48	− 2336.24	14029.7	− 7009.85	3270.169	− 1630.08	− 6504.17	3257.087	− 12114.7	6062.347	− 16695.7	8352.867	− 22764.8	11387.41
(0,1,4)	4274.759	− 2131.38	13726.77	− 6857.38	3200.903	− 1594.45	− 6504.53	3258.265	− 12118	6064.977	− 16713.2	8362.608	− 22795.6	11403.82
(1,1,0)	5329.461	− 2661.73	14998.12	− 7496.06	3293.819	− 1643.91	− 6509.97	3257.986	− 12125.6	6065.823	− 16725.2	8365.621	− 22824	11414.98
(1,1,1)	5252.667	− 2622.33	14975.92	− 7483.96	3282.417	− 1637.21	− 6509.11	3258.554	− 12124	6066.005	− 16729	8368.509	− 22827.3	11417.67
(1,1,2)	4308.619	− 2149.31	13822.36	− 6906.18	3278.778	− 1634.39	− 6507.26	3258.63	− 12126	6068.013	− 16733.2	8371.617	− 22839.1	11424.53
(1,1,3)	4235.198	− 2111.6	13773.48	− 6880.74	2787.171	− 1387.59	− 6505.4	3258.7	− 12124.3	6068.138	− 16732.4	8372.213	− 22841.3	11426.65
(1,1,4)	4215.811	− 2100.91	13727.8	− 6856.9	2766.9	− 1376.45	− 6503.65	3258.825	− 12122.5	6068.241	− 16731	8372.493	− 22839.9	11426.97
(2,1,0)	5110.534	− 2551.27	14854.46	− 7423.23	3275.18	− 1633.59	− 6509.14	3258.568	− 12124	6065.986	− 16728.4	8368.181	− 22826.6	11417.28
(2,1,1)	4229.131	− 2109.57	13784.48	− 6887.24	2766.557	− 1378.28	− 6507.19	3258.597	− 12125.6	6067.782	− 16729.6	8369.788	− 22830.7	11420.36
(2,1,2)	4231.111	− 2109.56	13773.13	− 6880.57	2756.909	− 1372.45	− 6505.19	3258.596	− 12124.6	6068.31	− 16731.9	8371.951	− 22841.4	11426.71
(2,1,3)	4226.22	− 2106.11	13761.2	− 6873.6	2757.024	− 1371.51	− 6503.37	3258.683	− 12122.6	6068.315	− 16730.2	8372.096	− 22839.5	11426.77
(2,1,4)	4216.028	− 2100.01	13725.9	− 6854.95	2758.625	− 1371.31	− 6501.48	3258.74	− 12121.2	6068.601	− 16728.4	8372.213	− 22839.1	11427.57
(3,1,0)	4887	− 2438.5	14603.33	− 7296.66	3238.141	− 1614.07	− 6507.25	3258.626	− 12126	6067.994	− 16733.4	8371.687	− 22837.8	11423.9
(3,1,1)	4231.107	− 2109.55	13767.88	− 6877.94	2758.079	− 1373.04	− 6505.2	3258.598	− 12124.3	6068.151	− 16732.8	8372.389	− 22841.9	11426.94
(3,1,2)	4233.045	− 2109.52	13769.09	− 6877.55	2756.771	− 1371.39	− 6503.21	3258.605	− 12123.1	6068.565	− 16732.7	8373.33	− 22839.9	11426.94
(3,1,3)	4234.906	− 2109.45	13746.25	− 6865.12	2759.46	− 1371.73	− 6501.35	3258.676	− 12121.2	6068.616	− 16730.6	8373.287	− 22837.6	11426.8
(3,1,4)	4215.044	− 2098.52	13727.25	− 6854.62	2760.782	− 1371.39	− 6735.56	3376.78	− 12119.3	6068.629	− 16727.4	8372.682	− 22837.5	11427.74
(4,1,0)	4756.607	− 2372.3	14345.71	− 7166.85	3199.712	− 1593.86	− 6505.64	3258.82	− 12124.1	6068.033	− 16733.6	8372.787	− 22839.4	11425.7
(4,1,1)	4225.01	− 2105.5	13767.01	− 6876.5	2758.179	− 1372.09	− 6503.48	3258.74	− 12122.3	6068.155	− 16731	8372.479	− 22839.9	11426.95
(4,1,2)	4235.095	− 2109.55	13757.57	− 6870.79	2760.302	− 1372.15	− 6501.42	3258.708	− 12121.3	6068.646	− 16729.3	8372.652	− 22838.5	11427.25
(4,1,3)	4219.256	− 2100.63	13738.25	− 6860.12	2759.809	− 1370.9	− 6714.39	3366.194	− 12124.2	6071.122	− 16729.4	8373.693	− 22837.7	11427.87
(4,1,4)	4217.068	− 2098.53	13726.44	− 6853.22	2764.489	− 1372.24	− 6725.16	3372.578	− 12118.7	6069.361	− 16727.5	8373.74	− 22835.4	11427.68

ARIMA Model	PRx ARIMA AIC	PRx ARIMA LL	L-PRx_5 ARIMA AIC	L-PRx_5 ARIMA LL	L-PRx_10 ARIMA AIC	L-PRx_10 ARIMA LL	L-PRx_20 ARIMA AIC	L-PRx_20 ARIMA LL	L-PRx_30 ARIMA AIC	L-PRx_30 ARIMA LL	L-PRx_40 ARIMA AIC	L-PRx_40 ARIMA LL	L-PRx_60 ARIMA AIC	L-PRx_60 ARIMA LL
Patient 5														
(0,1,0)	2033.231	− 1014.62	10215.49	− 5105.74	2980.582	− 1488.29	− 3566.58	1785.291	− 7601.98	3802.989	− 9970.42	4987.212	− 13857.6	6930.776
(0,1,1)	1905.645	− 949.822	10217.11	− 5105.56	2759.429	− 1376.71	− 4339.12	2172.558	− 8703.29	4354.646	− 11235.5	5620.762	− 15193.3	7599.664
(0,1,2)	1882.147	− 937.073	10028.89	− 5010.44	2748.376	− 1370.19	− 4482.13	2245.067	− 8956.11	4482.054	− 11509.4	5758.677	− 15515.3	7761.635
(0,1,3)	1619.001	− 804.501	9674.317	− 4832.16	2749.756	− 1369.88	− 4518.29	2264.147	− 9024.26	4517.132	− 11577	5793.487	− 15604.8	7807.384
(0,1,4)	1365.932	− 676.966	9424.486	− 4706.24	2733.425	− 1360.71	− 4517.05	2264.523	− 9045.55	4528.773	− 11600.3	5806.173	− 15642.2	7827.108
(1,1,0)	1929.741	− 961.871	10217.21	− 5105.6	2749.67	− 1371.83	− 4509.57	2257.786	− 9043.51	4524.756	− 11617.2	5811.602	− 15669.1	7837.536
(1,1,1)	1900.518	− 946.259	9551.458	− 4771.73	2751.353	− 1371.68	− 4510.11	2259.054	− 9047.5	4527.751	− 11616.7	5812.329	− 15676.8	7842.376
(1,1,2)	1307.386	− 648.693	9454.224	− 4722.11	2750.17	− 1370.08	− 4512.59	2261.295	− 9046.68	4528.341	− 11615	5812.485	− 15677.5	7843.774
(1,1,3)	1261.459	− 624.73	9438.409	− 4713.2	2404.936	− 1196.47	− 4516.86	2264.428	− 9046.19	4529.093	− 11613.2	5812.592	− 15678.3	7845.162
(1,1,4)	1250.714	− 618.357	9413.6	− 4699.8	2383.541	− 1184.77	− 4515.06	2264.528	− 9045.93	4529.967	− 11611.2	5812.614	− 15676.8	7845.403
(2,1,0)	1847.212	− 919.606	10138.17	− 5065.08	2751.205	− 1371.6	− 4510.5	2259.252	− 9047.84	4527.919	− 11616.6	5812.308	− 15676	7841.988
(2,1,1)	1255.104	− 622.552	9439.758	− 4714.88	2396.183	− 1193.09	− 4509.45	2259.724	− 9046.1	4528.048	− 11614.3	5812.134	− 15680.5	7845.259
(2,1,2)	1254.555	− 621.278	9435.056	− 4711.53	2368.281	− 1178.14	− 4700.91	2356.457	− 9044.07	4528.035	− 11612.8	5812.38	− 15673.7	7842.865
(2,1,3)	1253.29	− 619.645	9433.346	− 4709.67	2370.279	− 1178.14	− 4698.98	2356.489	− 9043.36	4528.681	− 11611.3	5812.64	− 15674.8	7844.378
(2,1,4)	1251.598	− 617.799	9411.701	− 4697.85	2369.73	− 1176.86	− 4514.46	2265.231	− 9043.78	4529.888	− 11609.3	5812.642	− 15674.6	7845.318
(3,1,0)	1723.582	− 856.791	10025.69	− 5007.85	2739.815	− 1364.91	− 4512.82	2261.41	− 9046.49	4528.246	− 11614.9	5812.463	− 15676.4	7843.196
(3,1,1)	1253.972	− 620.986	9433.338	− 4710.67	2372.312	− 1180.16	− 4700.05	2356.024	− 9044.11	4528.057	− 11612.7	5812.373	− 15678.5	7845.244
(3,1,2)	1255.229	− 620.615	9435.338	− 4710.67	2370.276	− 1178.14	− 4694.54	2354.268	− 9042.8	4528.398	− 11610.7	5812.326	− 15676.5	7845.259
(3,1,3)	1257.803	− 620.901	9436.41	− 4710.2	2371.793	− 1177.9	− 4703	2359.502	− 9163.03	4589.515	− 11609.2	5812.583	− 15674.6	7845.309
(3,1,4)	1241.864	− 611.932	9411.528	− 4696.76	2373.813	− 1177.91	− 4701.33	2359.665	− 9042.22	4530.11	− 11607.2	5812.593	− 15671.8	7844.888
(4,1,0)	1608.378	− 798.189	9841.442	− 4914.72	2712.353	− 1350.18	− 4522.16	2267.078	− 9046.35	4529.174	− 11613.2	5812.611	− 15676.9	7844.442
(4,1,1)	1252.893	− 619.447	9435.337	− 4710.67	2368.999	− 1177.5	− 4699.07	2356.533	− 9060.51	4537.254	− 11611.3	5812.628	− 15674.1	7844.033
(4,1,2)	1257.856	− 620.928	9427.368	− 4705.68	2374.878	− 1179.44	− 4703.7	2359.852	− 9162.01	4589.005	− 11609.2	5812.599	− 15674.6	7845.305
(4,1,3)	1239.891	− 610.946	9418.761	− 4700.38	2373.928	− 1177.96	− 4701.49	2359.744	− 9041.65	4529.826	− 11607.1	5812.574	− 15672.7	7845.363
(4,1,4)	1240.538	− 610.269	9412.944	− 4696.47	2376.069	− 1178.03	− 4693.2	2356.601	− 9159.17	4589.585	− 11605.2	5812.598	− 15670.8	7845.41

ARIMA Model	PRx ARIMA AIC	PRx ARIMA LL	L- PRx_5 ARIMA AIC	L- PRx_5 ARIMA LL	L- PRx_10 ARIMA AIC	L-PRx_10 ARIMA LL	L-PRx_20 ARIMA AIC	L-PRx_20 ARIMA LL	L-PRx_30 ARIMA AIC	L-PRx_30 ARIMA LL	L-PRx_40 ARIMA AIC	L-PRx_40 ARIMA LL	L-PRx_60 ARIMA AIC	L-PRx_60 ARIMA LL
Patient 6														
(0,1,0)	275.9623	− 135.981	16175.94	− 8085.97	4292.697	− 2144.35	− 5085.2	2544.599	− 10447.2	5225.578	− 14184.4	7094.204	− 19680.8	9842.39
(0,1,1)	261.7137	− 127.857	16100.91	− 8047.46	4247.607	− 2120.8	− 5465.04	2735.522	− 10977.7	5491.87	− 14854.1	7430.044	− 20579.7	10292.86
(0,1,2)	146.6525	− 69.3262	15751.96	− 7871.98	4248.88	− 2120.44	− 5514	2760.999	− 11052.8	5530.408	− 14935.9	7471.927	− 20652.5	10330.23
(0,1,3)	− 241.913	125.9565	15113.67	− 7551.83	4240.975	− 2115.49	− 5518.71	2764.353	− 11071	5540.492	− 14963.7	7486.86	− 20693.7	10351.87
(0,1,4)	− 496.606	254.303	14804.77	− 7396.39	4192.469	− 2090.23	− 5516.73	2764.363	− 11076.7	5544.326	− 14969	7490.501	− 20705.8	10358.89
(1,1,0)	265.272	− 129.636	16122.14	− 8058.07	4247.278	− 2120.64	− 5510.62	2758.309	− 11060.1	5533.028	− 14958.3	7482.144	− 20695.8	10350.9
(1,1,1)	247.3443	− 119.672	14842.92	− 7417.46	4249.257	− 2120.63	− 5518.92	2763.459	− 11082.2	5545.082	− 14979.2	7493.607	− 20701.5	10354.75
(1,1,2)	− 766.764	388.3819	14785.91	− 7387.96	4250.057	− 2120.03	− 5517.77	2763.886	− 11082	5545.989	− 14991.4	7500.715	− 20735	10372.52
(1,1,3)	− 786.477	399.2385	14766.23	− 7377.11	3611.898	− 1799.95	− 5516.56	2764.279	− 11080.1	5546.057	− 14989.4	7500.718	− 20733.3	10372.67
(1,1,4)	− 784.627	399.3133	14759.18	− 7372.59	3587.403	− 1786.7	− 5514.72	2764.362	− 11079	5546.504	− 14987.9	7500.927	− 20731.4	10372.68
(2,1,0)	157.3089	− 74.6544	15998.17	− 7995.08	4249.235	− 2120.62	− 5519.54	2763.768	− 11078.5	5543.26	− 14973.4	7490.698	− 20698.9	10353.43
(2,1,1)	− 786.773	398.3867	14777.39	− 7383.7	4251.273	− 2120.64	− 5517.41	2763.707	− 11081.8	5545.886	− 14989.8	7499.917	− 20724.3	10367.17
(2,1,2)	− 788.474	400.237	14765.66	− 7376.83	3561.925	− 1774.96	− 5515.44	2763.719	− 11079.8	5545.885	− 14989.4	7500.709	− 20733.3	10372.66
(2,1,3)	− 787.911	400.9554	14764.02	− 7375.01	3563.106	− 1774.55	− 5514.68	2764.338	− 11078	5545.996	− 14987.5	7500.754	− 20731.3	10372.63
(2,1,4)	− 786.261	401.1307	14757.07	− 7370.54	3563.867	− 1773.93	− 5512.72	2764.359	− 11076.5	5546.229	− 14985.8	7500.911	− 20729.3	10372.67
(3,1,0)	36.32196	− 13.161	15827.41	− 7908.71	4236.716	− 2113.36	− 5517.56	2763.78	− 11081.3	5545.641	− 14983.4	7496.701	− 20724.2	10367.12
(3,1,1)	− 788.417	400.2084	14763.01	− 7375.51	3583.418	− 1785.71	− 5515.54	2763.769	− 11080	5546.008	− 14989.7	7500.857	− 20733.2	10372.59
(3,1,2)	− 791.269	402.6347	14764.87	− 7375.43	3563.17	− 1774.58	− 5514.35	2764.177	− 11077.9	5545.965	− 14987.8	7500.876	− 20731.1	10372.56
(3,1,3)	− 789.797	402.8983	14769.54	− 7376.77	3565.885	− 1774.94	− 5512.82	2764.412	− 11076.2	5546.08	− 14985.6	7500.787	− 20729.3	10372.67
(3,1,4)	− 789.575	403.7876	14758.99	− 7370.49	3567.006	− 1774.5	− 5510.85	2764.426	− 11074.2	5546.119	− 14984.1	7501.033	− 20727.7	10372.87
(4,1,0)	− 29.3672	20.68362	15627.16	− 7807.58	4204.647	− 2096.32	− 5517.27	2764.635	− 11080.5	5546.262	− 14985.8	7498.922	− 20726.4	10369.19
(4,1,1)	− 786.42	400.2102	14764.64	− 7375.32	3566.972	− 1776.49	− 5515.11	2764.555	− 11078.4	5546.19	− 14988.6	7501.29	− 20731.1	10372.56
(4,1,2)	− 789.363	402.6814	14759.25	− 7371.63	3564.198	− 1774.1	− 5822.26	2919.13	− 11076.3	5546.165	− 14985.8	7500.902	− 20729.4	10372.71
(4,1,3)	− 793.399	405.6997	14759.83	− 7370.92	3567.077	− 1774.54	− 5849.58	2933.791	− 11074.3	5546.159	− 14982.4	7500.199	− 20727.1	10372.53
(4,1,4)	− 799.889	409.9443	14759.83	− 7369.92	3567.457	− 1773.73	− 5835.5	2927.751	− 11075.8	5547.894	− 14997.5	7508.754	− 20725.8	10372.91

ARIMA Model	PRx ARIMA AIC	PRx ARIMA LL	L-PRx_5 ARIMA AIC	L-PRx_5 ARIMA LL	L-PRx_10 ARIMA AIC	L-PRx_10 ARIMA LL	L-PRx_20 ARIMA AIC	L-PRx_20 ARIMA LL	L-PRx_30 ARIMA AIC	L-PRx_30 ARIMA LL	L-PRx_40 ARIMA AIC	L-PRx_40 ARIMA LL	L-PRx_60 ARIMA AIC	L-PRx_60 ARIMA LL
Patient 7														
(0,1,0)	− 1291.61	647.8046	14664.9	− 7330.45	4164.523	− 2080.26	− 3326.7	1665.348	− 7170.8	3587.401	− 10272.8	5138.415	− 14049.6	7026.782
(0,1,1)	− 1386.24	696.1184	14626.99	− 7310.5	4009.605	− 2001.8	− 3892.99	1949.495	− 7950.17	3978.083	− 11302.2	5654.075	− 15236	7620.996
(0,1,2)	− 1433.67	720.8334	14303.29	− 7147.65	4011.082	− 2001.54	− 3951.82	1979.908	− 8030.18	4019.089	− 11435.5	5721.757	− 15387.7	7697.828
(0,1,3)	− 1671.99	840.9941	13910.42	− 6950.21	4009.858	− 1999.93	− 3978.48	1994.24	− 8063.83	4036.914	− 11489.2	5749.601	− 15438.9	7724.474
(0,1,4)	− 1819.65	915.8237	13573.23	− 6780.62	3976.757	− 1982.38	− 3976.48	1994.24	− 8061.87	4036.933	− 11489	5750.488	− 15440	7725.985
(1,1,0)	− 1367.95	686.9758	14638.21	− 7316.11	4011.497	− 2002.75	− 3967.93	1986.964	− 8059.37	4032.685	− 11491.1	5748.55	− 15450.3	7728.147
(1,1,1)	− 1397.35	702.6759	13615.03	− 6803.51	4011.219	− 2001.61	− 3974.65	1991.325	− 8062.03	4035.013	− 11497.2	5752.593	− 15451	7729.483
(1,1,2)	− 2124.46	1067.232	13556.04	− 6773.02	4012.831	− 2001.42	− 3972.96	1991.482	− 8062.53	4036.263	− 11501.5	5755.748	− 15458.6	7734.304
(1,1,3)	− 2144.55	1078.275	13549.89	− 6768.95	3538.482	− 1763.24	− 3976.44	1994.22	− 8061.98	4036.989	− 11499.6	5755.776	− 15459.3	7735.642
(1,1,4)	− 2144.09	1079.047	13533.73	− 6759.87	3517.015	− 1751.51	− 3974.51	1994.254	− 8060.15	4037.074	− 11500.9	5757.437	− 15460.9	7737.467
(2,1,0)	− 1463.76	735.88	14518.06	− 7255.03	4010.263	− 2001.13	− 3973.87	1990.935	− 8061.48	4034.741	− 11496	5752.021	− 15450.6	7729.292
(2,1,1)	− 2142.43	1076.214	13550.96	− 6770.48	3534.903	− 1762.45	− 3972.81	1991.405	− 8061.84	4035.919	− 11501.2	5755.619	− 15460.2	7735.118
(2,1,2)	− 2140.52	1076.261	13549.46	− 6768.73	3508.587	− 1748.29	− 3975.48	1993.742	− 8067.46	4039.732	− 11504.7	5758.347	− 15454.7	7733.362
(2,1,3)	− 2154.63	1084.317	13549.42	− 6767.71	3508.869	− 1747.43	− 3975.65	1994.823	− 8065.38	4039.691	− 11504.5	5759.238	− 15454.7	7734.335
(2,1,4)	− 2153.67	1084.835	13529.53	− 6756.77	3508.451	− 1746.23	− 3973.25	1994.626	− 8064.5	4040.25	− 11503.7	5759.829	− 15464.6	7740.313
(3,1,0)	− 1574.6	792.2983	14388.42	− 7189.21	4002.95	− 1996.47	− 3974.31	1992.156	− 8062.93	4036.464	− 11500.2	5755.114	− 15455.1	7732.558
(3,1,1)	− 2140.55	1076.273	13548.47	− 6768.23	3516.783	− 1752.39	− 3976.8	1994.4	− 8067.53	4039.765	− 11503.6	5757.818	− 15452.8	7732.382
(3,1,2)	− 2149.7	1081.852	13554.94	− 6770.47	3509.209	− 1747.6	− 3975.43	1994.713	− 8065.1	4039.552	− 11506	5759.992	− 15464.1	7739.068
(3,1,3)	− 2153.95	1084.974	13550.96	− 6767.48	3511.639	− 1747.82	− 4224.38	2120.19	− 8063.53	4039.764	− 11503.9	5759.925	− 15462	7739.016
(3,1,4)	− 2152.05	1085.026	13519.69	− 6750.85	3512.401	− 1747.2	− 4231.06	2124.532	− 8062.73	4040.363	− 11502.2	5760.088	− 15463	7740.492
(4,1,0)	− 1624.31	818.1573	14134.04	− 7061.02	3972.429	− 1980.21	− 3976.86	1994.432	− 8062.08	4037.041	− 11498.2	5755.115	− 15453.8	7732.913
(4,1,1)	− 2144.11	1079.057	13550.4	− 6768.2	3507.143	− 1746.57	− 3975.12	1994.558	− 8065.59	4039.797	− 11503.3	5758.633	− 15451.1	7732.558
(4,1,2)	− 2153.63	1084.816	13542.89	− 6763.45	3508.754	− 1746.38	− 4216.73	2116.367	− 8063.85	4039.924	− 11504.1	5760.056	− 15462.1	7739.034
(4,1,3)	− 2152.91	1085.456	13529.09	− 6755.55	3512.235	− 1747.12	− 4232.33	2125.163	− 8062.22	4040.109	− 11502.2	5760.084	− 15460.7	7739.333
(4,1,4)	− 2151.72	1085.861	13519.11	− 6749.56	3512.931	− 1746.47	− 4223.17	2121.587	− 8061.41	4040.706	− 11504.3	5762.137	− 15461.4	7740.721

ARIMA Model	PRx ARIMA AIC	PRx ARIMA LL	L-PRx_5 ARIMA AIC	L-PRx_5 ARIMA LL	L-PRx_10 ARIMA AIC	L-PRx_10 ARIMA LL	L-PRx_20 ARIMA AIC	L-PRx_20 ARIMA LL	L-PRx_30 ARIMA AIC	L-PRx_30 ARIMA LL	L-PRx_40 ARIMA AIC	L-PRx_40 ARIMA LL	L-PRx_60 ARIMA AIC	L-PRx_60 ARIMA LL
Patient 8														
(0,1,0)	1000.719	− 498.36	14815.92	− 7405.96	3240.724	− 1618.36	− 5793.75	2898.874	− 10435.1	5219.54	− 13714.3	6859.153	− 19184.9	9594.475
(0,1,1)	913.5458	− 453.773	14803.42	− 7398.71	2974.14	− 1484.07	− 6682.29	3344.147	− 11714.7	5860.351	− 15255.4	7630.678	− 21128.7	10567.34
(0,1,2)	812.4078	− 402.204	14417.66	− 7204.83	2969.805	− 1480.9	− 6859.62	3433.81	− 11959.8	5983.913	− 15569.6	7788.781	− 21529.5	10768.77
(0,1,3)	381.0692	− 185.535	13855.83	− 6922.92	2965.595	− 1477.8	− 6886.77	3448.386	− 12022.4	6016.213	− 15652.7	7831.368	− 21721.1	10865.57
(0,1,4)	141.3732	− 64.6866	13586.65	− 6787.33	2920.021	− 1454.01	− 6899.41	3455.703	− 12047.1	6029.529	− 15687.6	7849.782	− 21748.2	10880.11
(1,1,0)	935.3981	− 464.699	14807.66	− 7400.83	2972.761	− 1483.38	− 6884.94	3445.472	− 12048	6027.017	− 15707	7856.497	− 21779.2	10892.58
(1,1,1)	891.7749	− 441.887	13710.06	− 6851.03	2971.72	− 1481.86	− 6898.98	3453.488	− 12060.7	6034.346	− 15720.7	7864.358	− 21791.2	10899.6
(1,1,2)	− 50.941	30.47051	13598.73	− 6794.36	2970.57	− 1480.28	− 6898.22	3454.11	− 12059.9	6034.972	− 15726.1	7868.063	− 21792.4	10901.19
(1,1,3)	− 76.8743	44.43713	13574.28	− 6781.14	2476.823	− 1232.41	− 6896.66	3454.331	− 12058.1	6035.055	− 15724.9	7868.443	− 21792.9	10902.47
(1,1,4)	− 75.6148	44.80739	13560.82	− 6773.41	2459.526	− 1222.76	− 6899.03	3456.517	− 12058.4	6036.199	− 15724.1	7869.055	− 21792.3	10903.15
(2,1,0)	789.3546	− 390.677	14677.54	− 7334.77	2970.107	− 1481.05	− 6899.85	3453.924	− 12059.6	6033.811	− 15718.7	7863.345	− 21790	10898.98
(2,1,1)	− 80.0325	45.01623	13582.06	− 6786.03	2469.846	− 1229.92	− 6897.9	3453.952	− 12059.6	6034.81	− 15725.5	7867.756	− 21791.1	10900.53
(2,1,2)	− 78.0344	45.01721	13577.05	− 6782.52	2449.91	− 1218.96	− 6896.01	3454.006	− 12057.7	6034.871	− 15724.5	7868.269	− 21801.1	10906.53
(2,1,3)	− 79.7321	46.86606	13571.66	− 6778.83	2451.747	− 1218.87	− 6894.24	3454.122	− 12055.9	6034.972	− 15723.1	7868.538	− 21799.2	10906.6
(2,1,4)	− 81.2009	48.60046	13562.1	− 6773.05	2452.743	− 1218.37	− 7150.85	3583.424	− 12055.3	6035.63	− 15721.1	7868.551	− 21797.1	10906.55
(3,1,0)	662.3942	− 326.197	14475.98	− 7232.99	2949.544	− 1469.77	− 6898.08	3454.038	− 12060.1	6035.031	− 15723.9	7866.948	− 21793.4	10901.68
(3,1,1)	− 78.035	45.01751	13573.74	− 6780.87	2452.414	− 1220.21	− 6896.03	3454.014	− 12057.5	6034.769	− 15724.7	7868.359	− 21801	10906.51
(3,1,2)	− 83.0737	48.53683	13575.19	− 6780.59	2451.802	− 1218.9	− 6894.01	3454.005	− 12055.8	6034.916	− 15721.7	7867.869	− 21798.9	10906.44
(3,1,3)	− 83.993	49.99649	13559.23	− 6771.61	2453.49	− 1218.74	− 7147.48	3581.74	− 12054.3	6035.13	− 15721.4	7868.685	− 21797.2	10906.6
(3,1,4)	− 83.0641	50.53203	13554.08	− 6768.04	2455.432	− 1218.72	− 7149.08	3583.541	− 12225.6	6121.821	− 15719.5	7868.746	− 21795.2	10906.6
(4,1,0)	564.8492	− 276.425	14242.99	− 7115.49	2914.164	− 1451.08	− 6896.62	3454.312	− 12058.3	6035.127	− 15726.2	7869.087	− 21793	10902.51
(4,1,1)	− 76.7053	45.35263	13573.48	− 6779.74	2451.087	− 1218.54	− 6894.13	3454.065	− 12056.4	6035.207	− 15724.2	7869.125	− 21799.2	10906.58
(4,1,2)	− 83.4488	49.7244	13577.21	− 6780.6	2454.841	− 1219.42	− 6892	3454.001	− 12054.5	6035.271	− 15721.8	7868.881	− 21797.1	10906.57
(4,1,3)	− 88.8822	53.44109	13556.06	− 6769.03	2455.359	− 1218.68	− 7148.9	3583.452	− 12063.1	6040.537	− 15719.2	7868.578	− 21795.1	10906.57
(4,1,4)	− 88.3128	54.15641	13555.19	− 6767.6	2457.375	− 1218.69	− 7145.18	3582.59	− 12061.4	6040.723	− 15726.8	7873.421	− 21794.2	10907.08

ARIMA Model	PRx ARIMA AIC	PRx ARIMA LL	L-PRx_5 ARIMA AIC	L-PRx_5 ARIMA LL	L-PRx_10 ARIMA AIC	L-PRx_10 ARIMA LL	L-PRx_20 ARIMA AIC	L-PRx_20 ARIMA LL	L-PRx_30 ARIMA AIC	L-PRx_30 ARIMA LL	L-PRx_40 ARIMA AIC	L-PRx_40 ARIMA LL	L-PRx_60 ARIMA AIC	L-PRx_60 ARIMA LL
Patient 9														
(0,1,0)	− 893.678	448.8392	11925.34	− 5960.67	3075.601	− 1535.8	− 3718.74	1861.369	− 7984.14	3994.068	− 11237.1	5620.527	− 14770.1	7387.054
(0,1,1)	− 1004.44	505.2178	11896.7	− 5945.35	2850.446	− 1422.22	− 4562.68	2284.338	− 9177.93	4591.967	− 12722	6364.017	− 16140.8	8073.417
(0,1,2)	− 1050.74	529.3712	11595.78	− 5793.89	2838.435	− 1415.22	− 4727.64	2367.821	− 9454.37	4731.183	− 13045.6	6526.816	− 16480.2	8244.107
(0,1,3)	− 1287.26	648.6323	11246.2	− 5618.1	2840.421	− 1415.21	− 4780.52	2395.26	− 9559.24	4784.622	− 13212.7	6611.344	− 16638.1	8324.047
(0,1,4)	− 1438.44	725.2221	10965.76	− 5476.88	2831.377	− 1409.69	− 4792.61	2402.306	− 9596.03	4804.015	− 13261.4	6636.711	− 16695	8353.494
(1,1,0)	− 980.35	493.175	11905.71	− 5949.85	2838.439	− 1416.22	− 4779.5	2392.751	− 9590.56	4798.282	− 13280.8	6643.388	− 16704.4	8355.216
(1,1,1)	− 1014.65	511.3239	11048.53	− 5520.26	2840.427	− 1416.21	− 4799.07	2403.536	− 9629.9	4818.948	− 13324.4	6666.218	− 16790.9	8399.466
(1,1,2)	− 1589.79	799.8925	10994.46	− 5492.23	2840.434	− 1415.22	− 4797.07	2403.535	− 9629.97	4819.987	− 13334.5	6672.262	− 16804.7	8407.352
(1,1,3)	− 1604.76	808.3779	10983.74	− 5485.87	2473.421	− 1230.71	− 4796.99	2404.495	− 9628.36	4820.18	− 13332.8	6672.408	− 16803	8407.505
(1,1,4)	− 1609.81	811.905	10962.35	− 5474.17	2447.767	− 1216.88	− 4795.75	2404.876	− 9626.74	4820.368	− 13331.5	6672.763	− 16801.4	8407.693
(2,1,0)	− 1088.51	548.2555	11805.71	− 5898.85	2840.422	− 1416.21	− 4798.22	2403.111	− 9625.31	4816.654	− 13315.8	6661.9	− 16771.3	8389.671
(2,1,1)	− 1596.54	803.2724	10988.6	− 5489.3	2842.327	− 1416.16	− 4797.07	2403.536	− 9629.61	4819.807	− 13332.5	6671.253	− 16804.8	8407.411
(2,1,2)	− 1598.55	805.2746	10987.95	− 5487.97	2422.813	− 1205.41	− 4795.27	2403.635	− 9628.04	4820.02	− 13333.1	6672.531	− 16803	8407.513
(2,1,3)	− 1605.45	809.7264	10984.23	− 5485.12	2838.848	− 1412.42	− 4794.31	2404.154	− 9626.13	4820.065	− 13331.2	6672.577	− 16800.7	8407.37
(2,1,4)	− 1621.84	818.92	10953.37	− 5468.68	2423.384	− 1203.69	− 4793.37	2404.683	− 9624.46	4820.228	− 13329.2	6672.624	− 16799.2	8407.582
(3,1,0)	− 1219.63	614.8169	11658.22	− 5824.11	2836.659	− 1413.33	− 4797.57	2403.783	− 9630.15	4820.077	− 13333.3	6671.658	− 16795.5	8402.739
(3,1,1)	− 1600.91	806.4571	10986.08	− 5487.04	2435.917	− 1211.96	− 4795.89	2403.944	− 9628.31	4820.156	− 13332.3	6672.158	− 16803.1	8407.537
(3,1,2)	− 1592.66	803.3286	10987.74	− 5486.87	2422.038	− 1204.02	− 4794.16	2404.078	− 9626.12	4820.059	− 13331.2	6672.608	− 16801.1	8407.533
(3,1,3)	− 1624.86	820.4278	10949.25	− 5466.63	2426.804	− 1205.4	− 4792.19	2404.094	− 9624.22	4820.11	− 13329.1	6672.574	− 16799.1	8407.532
(3,1,4)	− 1626.88	822.4421	10945.36	− 5463.68	2425.386	− 1203.69	− 5043.8	2530.902	− 9622.22	4820.109	− 13332.3	6675.164	− 16801.5	8409.742
(4,1,0)	− 1280.78	646.3913	11421.65	− 5704.82	2820.974	− 1404.49	− 4796.53	2404.263	− 9628.35	4820.176	− 13331.6	6671.814	− 16798.4	8405.192
(4,1,1)	− 1624.15	819.0752	10985.38	− 5485.69	2425.571	− 1205.79	− 4794.14	2404.068	− 9626.31	4820.157	− 13329.3	6671.66	− 16801.1	8407.536
(4,1,2)	− 1630.24	823.1196	10977.86	− 5480.93	2423.38	− 1203.69	− 4792.14	2404.072	− 9624.34	4820.168	− 13329	6672.479	− 16799.1	8407.545
(4,1,3)	− 1627.66	822.8313	10952.03	− 5467.01	2426.012	− 1204.01	− 4790.69	2404.347	− 9622.23	4820.116	− 13329.1	6673.552	− 16798.2	8408.078
(4,1,4)	− 1622.65	821.3236	10933.41	− 5456.7	2423.177	− 1201.59	− 5036.68	2528.339	− 9620.25	4820.125	− 13330.3	6675.172	− 16799.3	8409.666

ARIMA Model	PRx ARIMA AIC	PRx ARIMA LL	L-PRx_5 ARIMA AIC	L-PRx_5 ARIMA LL	L-PRx_10 ARIMA AIC	L-PRx_10 ARIMA LL	L-PRx_20 ARIMA AIC	L-PRx_20 ARIMA LL	L-PRx_30 ARIMA AIC	L-PRx_30 ARIMA LL	L-PRx_40 ARIMA AIC	L-PRx_40 ARIMA LL	L-PRx_60 ARIMA AIC	L-PRx_60 ARIMA LL
Patient 10														
(0,1,0)	163.2518	− 79.6259	16909.69	− 8452.85	4185.217	− 2090.61	− 6593.55	3298.773	− 12139.9	6071.97	− 16121.9	8062.971	− 22740.4	11372.2
(0,1,1)	43.72775	− 18.8639	16898.26	− 8446.13	3867.44	− 1930.72	− 7463.4	3734.698	− 13386.5	6696.245	− 17645.8	8825.923	− 24607.6	12306.8
(0,1,2)	− 25.7855	16.89274	16536.7	− 8264.35	3851.529	− 1921.76	− 7639.01	3823.507	− 13701.8	6854.914	− 18070.7	9039.346	− 25200.9	12604.43
(0,1,3)	− 336.423	173.2117	15919.33	− 7954.67	3850.324	− 1920.16	− 7700.07	3855.035	− 13812.2	6911.093	− 18219.4	9114.681	− 25401.9	12705.96
(0,1,4)	− 538.916	275.4581	15490.67	− 7739.34	3825.536	− 1906.77	− 7713.06	3862.532	− 13854.5	6933.25	− 18303.1	9157.558	− 25493.1	12752.56
(1,1,0)	68.73661	− 31.3683	16901.95	− 8447.98	3854.824	− 1924.41	− 7668.67	3837.335	− 13792.1	6899.037	− 18227.1	9116.547	− 25458.7	12732.35
(1,1,1)	26.97656	− 9.48828	15638.97	− 7815.49	3856.429	− 1924.21	− 7706.84	3857.419	− 13868.1	6938.053	− 18322.7	9165.332	− 25597.1	12802.56
(1,1,2)	− 850.326	430.1628	15512.38	− 7751.19	3852.383	− 1921.19	− 7706.38	3858.189	− 13866.6	6938.312	− 18321	9165.475	− 25595.7	12802.85
(1,1,3)	− 871.22	441.61	15486.82	− 7737.41	3199.152	− 1593.58	− 7711.29	3861.644	− 13868.1	6940.072	− 18320.9	9166.455	− 25595.3	12803.63
(1,1,4)	− 870.017	442.0083	15458	− 7722	3166.209	− 1576.1	− 7711.74	3862.869	− 13869.2	6941.622	− 18322.7	9168.361	− 25593.3	12803.64
(2,1,0)	− 61.1811	34.59056	16765.55	− 8378.78	3856.223	− 1924.11	− 7707.21	3857.606	− 13865.3	6936.659	− 18318.7	9163.355	− 25586.6	12797.29
(2,1,1)	− 867.929	438.9643	15493.91	− 7741.96	3858.71	− 1924.35	− 7705.86	3857.929	− 13866.5	6938.255	− 18320.9	9165.452	− 25595.8	12802.9
(2,1,2)	− 866.928	439.4638	15485.8	− 7736.9	3128.724	− 1558.36	− 7703.02	3857.51	− 13864.1	6938.063	− 18318.8	9165.388	− 25593.1	12802.57
(2,1,3)	− 871.67	442.8349	15481.13	− 7733.56	3130.03	− 1558.01	− 8089.1	4051.55	− 13864.1	6939.037	− 18317	9165.517	− 25592.4	12803.19
(2,1,4)	− 872.245	444.1227	15456.91	− 7720.46	3194.675	− 1589.34	− 7709.73	3862.865	− 13866	6940.988	− 18321.4	9168.703	− 25591.3	12803.66
(3,1,0)	− 203.388	106.6938	16569.07	− 8279.53	3834.951	− 1912.48	− 7706.43	3858.216	− 13867.5	6938.766	− 18321.5	9165.775	− 25592.6	12801.3
(3,1,1)	− 867.261	439.6304	15482.49	− 7735.25	3141.562	− 1564.78	− 7705.22	3858.611	− 13865.7	6938.858	− 18319.5	9165.775	− 25594.8	12803.38
(3,1,2)	− 865.793	439.8965	15484.45	− 7735.22	3130.098	− 1558.05	− 8078.85	4046.427	− 13863.8	6938.881	− 18317.1	9165.528	− 25592.5	12803.27
(3,1,3)	− 871.288	443.6442	15486.16	− 7735.08	3131.172	− 1557.59	− 8082.56	4049.282	− 14124.2	7070.115	− 18314.8	9165.391	− 25590.7	12803.34
(3,1,4)	− 870.108	444.0539	15448.09	− 7715.05	3130.903	− 1556.45	− 8085.61	4051.806	− 14127.3	7072.663	− 18319.5	9168.749	− 25589.7	12803.85
(4,1,0)	− 299.205	155.6023	16282.85	− 8135.43	3800.071	− 1894.04	− 7710.49	3861.243	− 13866.2	6939.08	− 18319.6	9165.777	− 25594.4	12803.18
(4,1,1)	− 875.543	444.7717	15484.29	− 7735.14	3131.513	− 1558.76	− 8083.46	4048.728	− 13863.7	6938.852	− 18317.9	9165.933	− 25593.3	12803.66
(4,1,2)	− 873.137	444.5686	15475.76	− 7729.88	3131.299	− 1557.65	− 8086.99	4051.495	− 13862.2	6939.108	− 18320.3	9168.171	− 25590.9	12803.45
(4,1,3)	− 870.12	444.06	15450.62	− 7716.31	3131.424	− 1556.71	− 8085.2	4051.599	− 14127.5	7072.729	− 18319	9168.492	− 25589.8	12803.9
(4,1,4)	− 888.688	454.3438	15444.3	− 7712.15	3135.845	− 1557.92	− 8081.45	4050.724	− 14124.4	7072.187	− 18317.5	9168.741	− 25587.3	12803.66

Optimal ARIMA models are determined by lowest AIC and highest LL

**Table 5:** Patient example—ARIMA model AIC and LL for ICP and MAP—using 10-s and minute averaged data

ARIMA Model	10-s mean data				Minute mean data			
	ICP AIC	ICP LL	MAP AIC	MAP LL	ICP AIC	ICP LL	MAP AIC	MAP LL
(0,1,0)	196388.8	− 98192.4	269930.5	− 134963	39569.24	− 19782.6	71689.08	− 35842.5
(0,1,1)	196368.8	− 98181.4	265609.2	− 132802	38779.78	− 19386.9	68214.99	− 34104.5
(0,1,2)	192234.3	− 96113.1	265437.9	− 132715	38678.96	− 19335.5	68141.03	− 34066.5
(0,1,3)	190875.4	− 95432.7	265199.9	− 132595	38608.42	− 19299.2	68085.34	− 34037.7
(0,1,4)	190771.8	− 95379.9	265201.8	− 132595	38571.06	− 19279.5	68023.08	− 34005.5
(1,1,0)	196379	− 98186.5	266268.8	− 133131	38960.74	− 19477.4	69664.86	− 34829.4
(1,1,1)	192440.5	− 96216.3	265351.4	− 132672	38589.53	− 19290.8	68118.71	− 34055.4
(1,1,2)	190892.8	− 95441.4	265305	− 132647	38549.22	− 19269.6	67983.68	− 33986.8
(1,1,3)	190791	− 95389.5	265201.9	− 132595	38551.12	− 19269.6	67985.65	− 33986.8
(1,1,4)	190770	− 95378	265203.7	− 132595	38478.1	− 19232.1	67987.03	− 33986.5
(2,1,0)	193682.1	− 96837	265874.4	− 132933	38830.76	− 19411.4	68976.31	− 34484.2
(2,1,1)	190776.9	− 95383.5	265278.4	− 132634	38549.59	− 19269.8	68038.36	− 34014.2
(2,1,2)	190778.9	− 95383.4	265220.9	− 132604	38551.23	− 19269.6	67985.63	− 33986.8
(2,1,3)	190772.4	− 95379.2	265160.8	− 132573	38552.94	− 19269.5	67987.45	− 33986.7
(2,1,4)	190757	− 95370.5	265150.6	− 132567	38554.87	− 19269.4	67989.04	− 33986.5
(3,1,0)	192186.7	− 96088.3	265385.2	− 132688	38753.15	− 19371.6	68750.63	− 34370.3
(3,1,1)	190778.8	− 95383.4	265187.1	− 132588	38551.15	− 19269.6	67991.69	− 33989.8
(3,1,2)	190780.8	− 95383.4	265146.3	− 132566	38553.44	− 19269.7	67988.98	− 33987.5
(3,1,3)	190781.7	− 95382.8	265148.3	− 132566	38549.55	− 19266.8	67988.83	− 33986.4
(3,1,4)	190739.8	− 95360.9	265140.9	− 132561	38557.21	− 19269.6	67989.47	− 33985.7
(4,1,0)	191405.4	− 95696.7	265215.2	− 132602	38689.2	− 19338.6	68607.28	− 34297.6
(4,1,1)	190727.9	− 95357	265174.7	− 132580	38553.08	− 19269.5	67991.2	− 33988.6
(4,1,2)	190781.3	− 95382.7	265148.7	− 132566	38555.06	− 19269.5	67993.81	− 33988.9
(4,1,3)	190783.9	− 95383	265146.6	− 132564	38549.9	− 19265.9	67982.67	− 33982.3
(4,1,4)	190449.4	− 95214.7	265142.4	− 132561	38549.27	− 19264.6	67991.68	− 33985.8

Comparing the optimal AIC and LL for ICP and MAP, between 10-s and minute mean data, there is no substantial difference in the time series structure. This pattern was found in all 31 of the patients included in this study, with this patient’s data displayed as an example

*AIC* akaike information criterion, *ARIMA* autoregressive integrative moving average, *ICP* intracranial pressure, *MAP* mean arterial pressure

**Table 6:** Univariate logistic regression analysis -cerebrovascular reactivity and dichotomized outcome at 6 months

Model	AUC A/D	95% CI	AIC	P-value	AUC F/U	95% CI	AIC	P-value
PRx	0.784	0.520–0.986	26.0	0.02	0.418	0.222–0.644	49.2	0.552
L-PRx_5	0.832	0.624–0.992	24.1	0.007	0.587	0.369–0.791	49.0	0.475
L-PRx_10	0.880	0.728–0.999	23.2	0.005	0.591	0.369–0.796	48.9	0.446
L-PRx_20	0.880	0.728–0.992	22.4	0.003	0.609	0.391–0.800	48.7	0.378
L-PRx_30	0.888	0.736–0.999	22.6	0.003	0.627	0.409–0.818	48.7	0.367
L-PRx_40	0.880	0.712–0.992	22.8	0.004	0.613	0.404–0.813	48.6	0.357
L-PRx_60	0.848	0.688–0.968	23.3	0.005	0.596	0.378–0.796	48.6	0.344

AUC and 95% CI calculated using bootstrap methodology with 2000 iterations. Bolded values indicate those reaching statistical significance (alpha of 0.05)

*A/D* alive/dead, *AUC* area under the receiver operating curve, *CI* confidence interval, *F/U* favourable/unfavourable outcome (i.e. favourable = glasgow outcome scale of 5 to 8; unfavourable = glasgow outcome scale of 1 to 4), *ICP* intra-cranial pressure, *MAP* mean arterial pressure, *PRx* pressure reactivity index (correlation between ICP and MAP)
